# Physiologically-Based Toxicokinetic Modeling of Zearalenone and Its Metabolites: Application to the Jersey Girl Study

**DOI:** 10.1371/journal.pone.0113632

**Published:** 2014-12-04

**Authors:** Dwaipayan Mukherjee, Steven G. Royce, Jocelyn A. Alexander, Brian Buckley, Sastry S. Isukapalli, Elisa V. Bandera, Helmut Zarbl, Panos G. Georgopoulos

**Affiliations:** 1 Environmental and Occupational Health Sciences Institute, Rutgers University, Piscataway, New Jersey, United States of America; 2 Department of Environmental and Occupational Medicine, Rutgers University - Robert Wood Johnson Medical School, Piscataway, New Jersey, United States of America; 3 Department of Chemical and Biochemical Engineering, Rutgers University, Piscataway, New Jersey, United States of America; 4 Rutgers Cancer Institute of New Jersey, Rutgers University, New Brunswick, New Jersey, United States of America; Utah State University, United States of America

## Abstract

Zearalenone (ZEA), a fungal mycotoxin, and its metabolite zeranol (ZAL) are known estrogen agonists in mammals, and are found as contaminants in food. Zeranol, which is more potent than ZEA and comparable in potency to estradiol, is also added as a growth additive in beef in the US and Canada. This article presents the development and application of a Physiologically-Based Toxicokinetic (PBTK) model for ZEA and ZAL and their primary metabolites, zearalenol, zearalanone, and their conjugated glucuronides, for rats and for human subjects. The PBTK modeling study explicitly simulates critical metabolic pathways in the gastrointestinal and hepatic systems. Metabolic events such as dehydrogenation and glucuronidation of the chemicals, which have direct effects on the accumulation and elimination of the toxic compounds, have been quantified. The PBTK model considers urinary and fecal excretion and biliary recirculation and compares the predicted biomarkers of blood, urinary and fecal concentrations with published *in vivo* measurements in rats and human subjects. Additionally, the toxicokinetic model has been coupled with a novel probabilistic dietary exposure model and applied to the Jersey Girl Study (JGS), which involved measurement of mycoestrogens as urinary biomarkers, in a cohort of young girls in New Jersey, USA. A probabilistic exposure characterization for the study population has been conducted and the predicted urinary concentrations have been compared to measurements considering inter-individual physiological and dietary variability. The *in vivo* measurements from the JGS fall within the high and low predicted distributions of biomarker values corresponding to dietary exposure estimates calculated by the probabilistic modeling system. The work described here is the first of its kind to present a comprehensive framework developing estimates of potential exposures to mycotoxins and linking them with biologically relevant doses and biomarker measurements, including a systematic characterization of uncertainties in exposure and dose estimation for a vulnerable population.

## Background

Growth-promoting hormones are produced naturally in the animal body and can also be artificially synthesized and supplemented. There are about 30 growth-promoting hormonal products currently used in the United States [1], the most important being estradiol, progesterone, testosterone, zeranol, trenbolone acetate, diethylstilbestrol (DES) and melengestrol acetate [1]. They are used primarily in animal husbandry to improve meat output and increase feed-to-meat conversion. Zeranol (ZAL), an estrogenic compound derived from fungi of the *Fusarium* family, is used as a growth-promoter in cattle in many countries, including Canada, Australia, New Zealand, South Africa, Mexico, Chile, Japan, and the US. In the US, it is used under the trade name *Ralgro* as an anabolic agent for more efficient conversion of feed to meat. The use of synthetic growth-promoters such as zeranol is controversial due to their ability to mimic actions of indigenous hormones of the animal body, potentially leading to abnormal outcomes. Zeranol is a known estrogen-agonist and its estrogenicity has been found to be comparable to that of natural estradiol [2]. The parent compound of zeranol, zearalenone (ZEA) is a common fungal contaminant found in a number of cereal crops, fruits, and vegetables across the world [3]. ZEA, ZAL and their metabolites have been implicated in a number of incidences involving precocious puberty among young girls in various countries, including Italy in 1979 [4], and Puerto Rico in the 1980s [5]–[7], although actual levels were not measured. Massart *et al.* [8] reported ZEA to be associated with central precocious puberty (CPP) in young females in a study of 63 girls in Italy, however, important covariates were not taken into account. However, the risks associated with dietary exposure to ZAL residues in beef have not yet been conclusively characterized, with various agencies publishing conflicting reports regarding the long-term effects of consuming meat treated with such synthetic hormones [1]. Zeranol, along with DES and Melengestrol Acetate, have been banned as meat additives in the European Union (EU) [9], further leading to an EU ban on the import of meat products from the US and Canada. The dietary exposure and the metabolism and clearance of these compounds in living systems is crucial to understanding their effects on sexual development. The Joint FAO/WHO Expert Committee on Food Additives (JECFA) sets the Allowable Daily Intake (ADI) of ZAL for humans at 0.5 *µ*g per kg body weight [10], which amounts to a 35 *µ*g allowable daily intake for a 70 kg male.

### Exposure and Biotransformation

**Figure 1:**
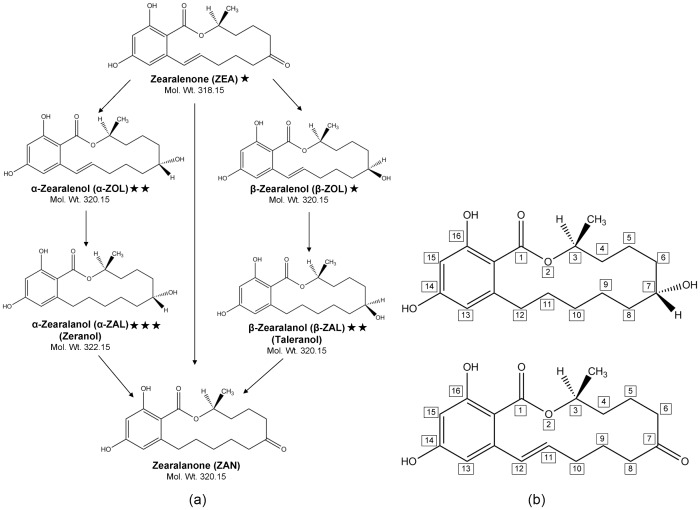
Chemical structures of ZEA and its metabolites. (a) Compounds of the zeranol family of mycotoxins and their biotransformation pathways (adapted from Kleinova *et al.* [29]); The stars represent the relative estrogenic potency of the compounds which is in the order: *α*-ZAL> *α*-ZOL> *β*-ZAL> ZEA> *β*-ZOL. (b) Zearalenone (ZEA) (bottom) and Zeranol (ZAL) (top) molecules with their structural representation.

**Figure 2:**
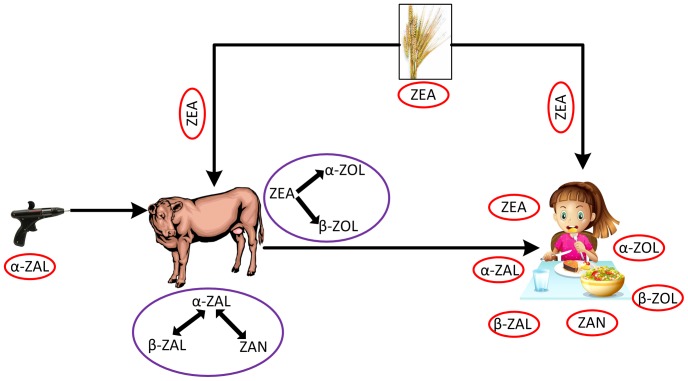
Human exposure. Human exposure routes of zearalenone and zeranol.

Alpha-zearalanol (*α*-ZAL), commonly known as zeranol, is a fungal metabolite produced from zearalenone (ZEA), a non-steroidal estrogenic mycotoxin. Fig. 1(a) shows the various compounds of the zeranol family. The study of biotransformation of this family of compounds has been the focus of world-wide interest due to their estrogenic function. Humans are exposed to both ZEA and *α*-ZAL mainly through consumption of contaminated grains [11], though there have been examples of other routes of exposure such as inhalation exposure in farm workers [12], [13]. However, the most important exposure pathway for *α*-ZAL can be meat treated with it as a growth additive. *α*-ZAL is administered to livestock in the form of 12 mg pellets, and a dose of 3 pellets (36 mg) is considered to be sufficient for 90-120 days [14]. Human exposure to *α*-ZAL might also result from consumption of farm animals which have consumed and metabolized ZEA-contaminated fodder. Fig. 2 shows the various exposure pathways resulting to human intake of ZEA and ZAL.

**Figure 3:**
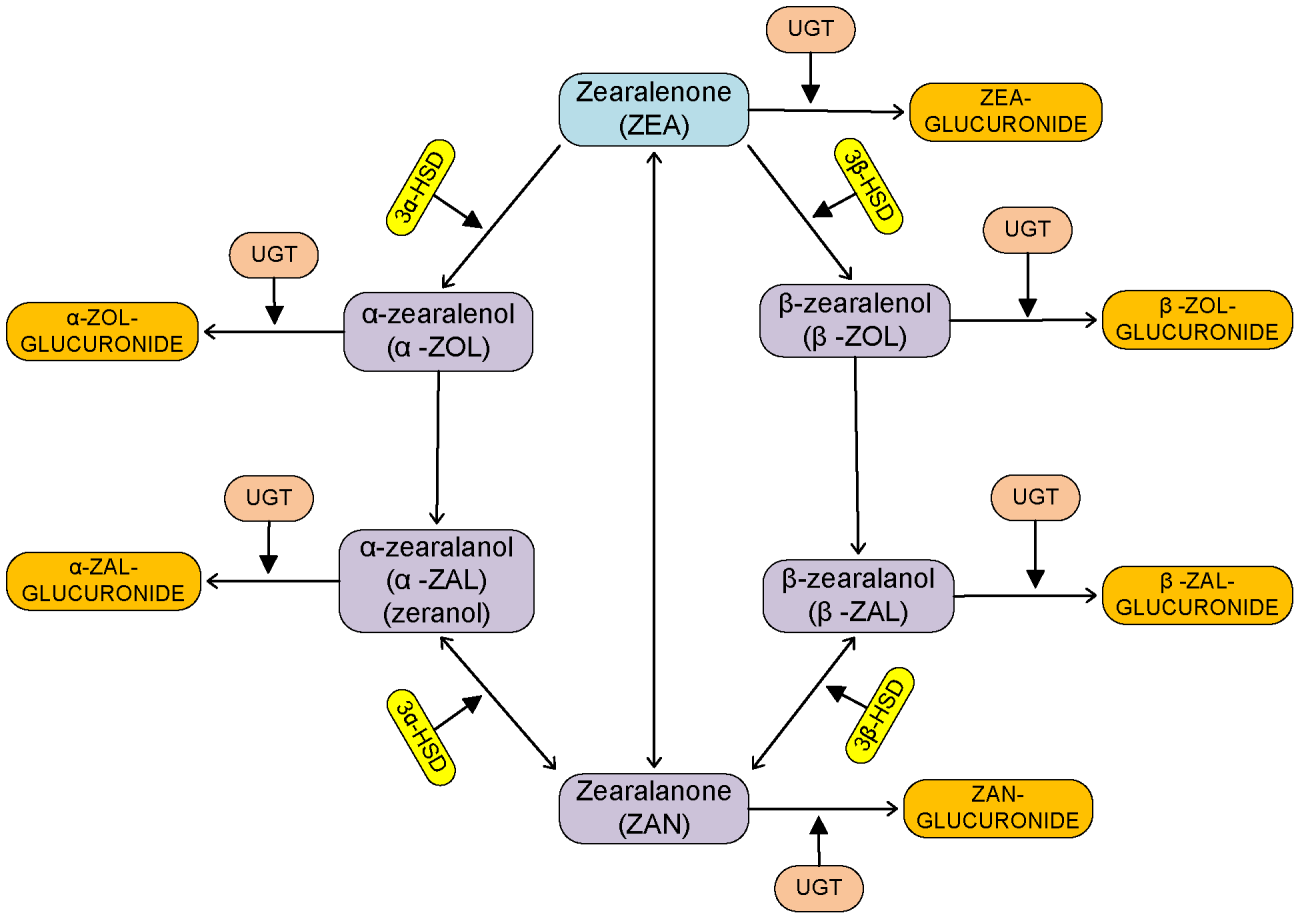
ZEA metabolism. Major biotransformation pathways of zearalenone and zeranol in mammals.

The first analysis of the *in vivo* metabolism of ZEA was done by Kiessling & Pettersson [15], who studied the livers of rats dosed with ZEA, and identified two major phases of metabolism: the reduction of the ketone group of zearalenone to zearalenol (ZOL), and the conjugation by glucuronic acid to form glucuronide. These two phases of biotransformation of ZEA have been subsequently confirmed by many researchers such as Migdalof *et al.* [16], Fitzpatrick *et al.* [17], and Bories *et al.* [18] and are generally regarded as Phase I and Phase II metabolic processes. Fitzpatrick *et al.* [17] analyzed the metabolism of zearalenone and found *α*-zearalenol to be the major metabolite in the feces and the conjugated glucuronides to be the major metabolites in the urine. Migdalof *et al.* [16] found zearalanone (ZAN) and some conjugated glucuronides in the urine of various species after ingestion of zeranol. They also found some unknown metabolites in the urine, which were later confirmed to be catechols formed by aromatic hydroxylation on the zearalenone phenolic ring [19]. Fig. 3 summarizes the major metabolic pathways of zearalenone and zearalenol. The biotransformation of zearalenone or zeranol, especially the conversion extents to various metabolites formed, were, however, found to vary widely across species, making the task of inter-species scaling very difficult. The extents and rates of various metabolic reactions are important because the metabolites of ZEA vary in their estrogenic potential. The estrogenicity of the compounds has been found to be in the order: *α*-ZAL> *α*-ZOL> *β*-ZAL> ZEA> *β*-ZOL.

#### Phase-I metabolism

Aliphatic hydroxylation reduces the ketone group in ZEA or ZAN to form the corresponding alcohol. ZEA is converted to *α*-, *β*-zearalenol (ZOL) and this conversion is catalyzed by 3 *α*-, and 3 *β*-hydroxy steroid dehydrogenase (HSD) [20]. Both Pfeiffer *et al.* [19] and Malekinejad *et al.* [21] have found a considerable variance of the reaction rates across species. For example, rats were found to produce more of *β*-ZOL, while pigs and humans have a definite preference towards *α*-ZOL [21]. The interspecies differences in metabolism lead to varying estrogenic effects in various animals, the pig being most sensitive. Aromatic hydroxylation adds a hydroxyl group to the aromatic ring at the available 13 or 15 positions, forming 13- and 15-hydroxy ZEN or ZAN. This transformation has been found to be catalyzed by cytochrome P450 (CYP450) enzymes [19]. The major enzyme isoforms responsible for this process are CYP1A2, CYP1B1, CYP2D6, CYP2C19 and CYP3A4. The 13- and 15-catechol metabolites were studied extensively by Pfeiffer *et al.* [19]. The 15-hydroxy catechol is more predominant, in both rat and human, than the 13 isomer, probably because of the steric hindrance to addition at the 13 position. These catechols are transformed to their mono-methyl esters by catechol-o-methyl transferase (COMT) and S-adenosyl methionine (SAM) [22]. These metabolites may eventually get converted to quinones [23] and lead to formation of reactive oxygen species and cause DNA adducts [24], [25].

#### Phase-II metabolism

This phase of metabolism concerns the glucuronidation and sulfation of the parent compounds or the phase I metabolites. The glucuronic acid group is supplied by uridine 5′-diphosphate glucuronic acid (UDPGA) and the conversion is catalyzed by uridine 5′-diphosphate glucuronosyltransferase (UGT) [26]. Pfeiffer *et al.* [26] found that UGT1A1, 1A3, 1A8 and 2B7 seem to be the most active isoforms and they compiled the kinetic constants for different species. Glucuronidation has been identified as the major phase II metabolic process in animals [19], [21]. Sulfation, a minor conjugation pathway has not been considered in this model. (While Fig. 3 summarizes the major biotransformation pathways of zearalenone, zearalanol and related compounds, Figure S1 in S1 Information shows a more comprehensive pathway network of possible ZEA biotransformation processes based on information from the literature.)

### Excretion

The mode of excretion of zeranol is also widely variable among species. Humans excrete zeranol mostly by urine [27] whereas for other species, such as dogs and rats, fecal elimination is the major route of excretion. Migdalof *et al.* [16] found zearalanone (ZAN) to be a major and taleranol (*β*-zeranol) a minor metabolite of zeranol in humans. However, no traces of ZEA or ZOLs (*α*- or *β*-ZOL) were found in mammals after dosing with *α*-ZAL. This suggests that zeranol and taleranol have a reversible relation with ZAN but the ZALs are not converted to their unsaturated forms in mammals. Kiessling & Pettersson [15] showed that rats do not produce zeranol or taleranol when dosed with zearalenone. However, Miles *et al.* [28] found zeranol metabolites in sheep dosed with zearalenone. Similar studies were conducted for heifers [29] and pigs [30], where zeranol and taleranol, among other metabolites, were found in the urine of animals fed ZEA-contaminated cereals. In light of these studies, it may be assumed that though the reduction of *α*- and *β*-ZOL and of ZEA to ZALs and ZAN takes place negligibly in mammalian systems (none in rats), there is no evidence at present that the reverse occurs.

## Methods

### PBTK modeling of ZEA and its metabolites

Our main goal in this study is to formulate a comprehensive Physiologically Based Toxicokinetic (PBTK) model for realistic exposures to ZEA and ZAL from either fungal-infected food grains or from growth-promoter added meat, that will quantify mechanistically the bio-transformation, metabolism and excretion of all related compounds. The only existing PBTK model for ZEA was developed by Shin *et al.* [31] for rats in conjunction with *in vivo* experiments with intravenous and oral dosing. The PBTK model was subsequently scaled up to humans by Shin *et al.* using allometric scaling according to body weight. However, the model does not consider zeranol or any other metabolite of ZEA. Because of the variable estrogenic potencies of the different metabolites, a comprehensive risk analysis should quantify the various metabolites in the body. The model by Shin *et al.* also does not include urine or feces which are used as biomarkers to monitor exposure and biological clearance rates in animals. The difference in estrogenicities among the metabolites coupled with the variations in their residence times within the body could affect the long term risks of exposed individuals.

### Features of the PBTK model

**Figure 4:**
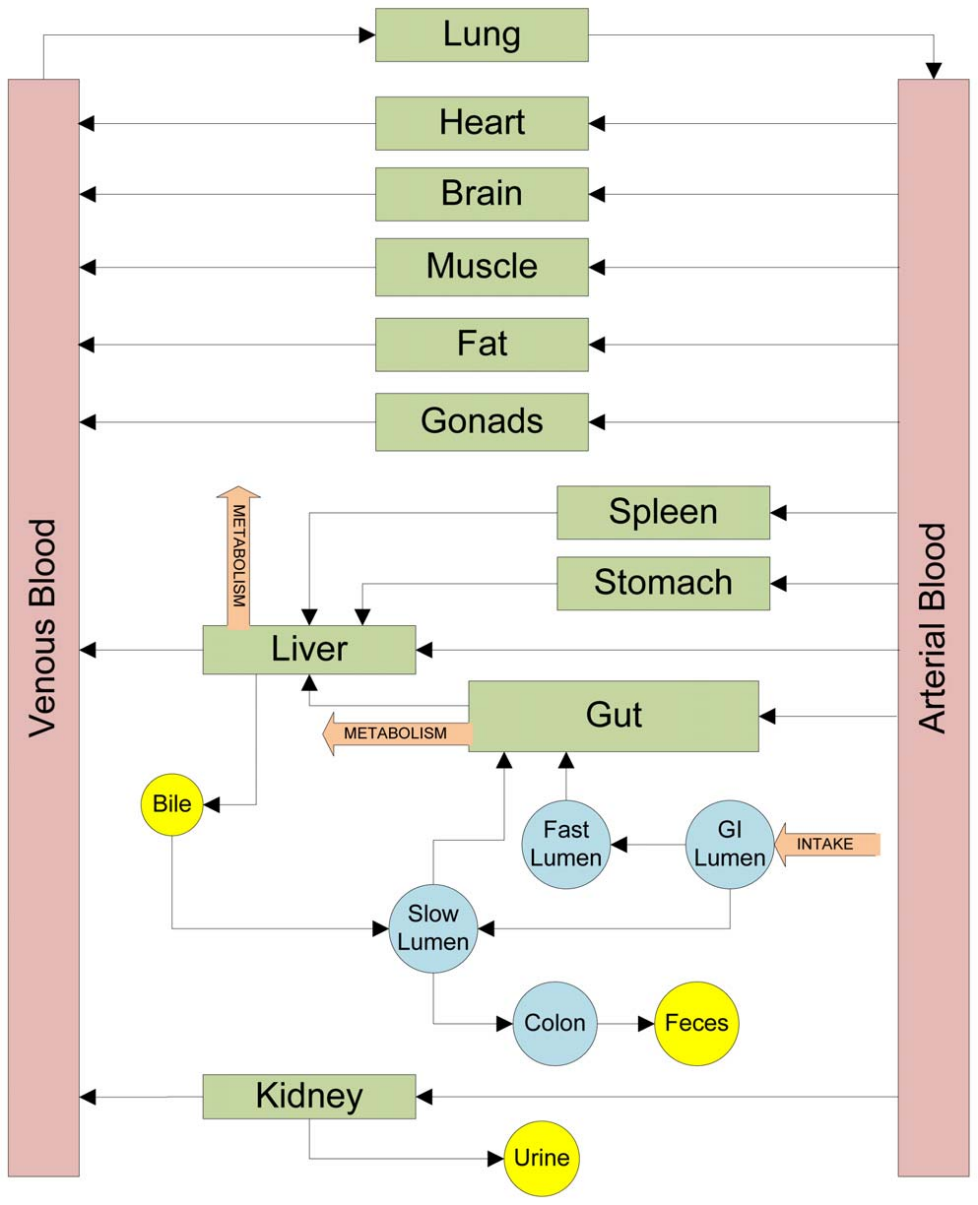
Model structure. Structure of the PBTK model developed for human subjects.

The PBTK model developed here expands upon the physiological compartments considered by Shin *et al.* [31] and introduces additional physiologically and toxicologically relevant entities, including urine, feces and bile, which can help utilize biomarker data and account for various metabolic endpoints. ZEA, ZAL, their various hydrogenated metabolites and their conjugates are separately considered in this model. Absorption dynamics in the gut and entero-hepatic recirculation of parent compounds and their metabolites are also explicitly modeled. ZEA is known to undergo phase-I and phase-II metabolism both in the liver and in the gut (discussed in the “Exposure and Biotransformation” section) and considerable inter-species differences have been reported in the formation of various metabolites and in their ultimate fate in the body [16], [21]. The model accounts for phase-I and phase-II metabolism separately and considers the metabolites *α*-ZOL, and *β*-ZOL of ZEA and *α*-ZAL, *β*-ZAL, and ZAN of zeranol and their respective glucuronides (ZEAGLU, *α*-ZOLGLU, *β*-ZOLGLU, etc.). Extra-hepatic metabolism of ZEA, especially in the gut, is found to be of considerable importance in the study of ZEA biodistribution [32], [33]. The PBTK model considers gut metabolism (both phase-I and phase-II) of ZEA and ZAL in both rat and human. A model for rats is presented in this section, followed by a whole body PBTK model for human subjects in the section: “Extension of the PBTK model to human subjects”. A total of twelve compounds (six metabolites and their respective conjugated glucuronides) have been considered in this PBTK model and their transport, absorption and mutual bio-transformation has been computed. The twelve compounds are denoted by indices 1 through 12. The rat PBTK model helps validate various mechanistic processes that have been considered. Fig. 4 gives a schematic representation of the model developed here. The balance of all twelve chemicals across the physiological compartments is represented by the following equations, where, subscripts *i* = 1 to 12 denote ZEA, *α*-ZOL, *β*-ZOL, *α*-ZAL, *β*-ZAL, and ZAN and their glucuronidated conjugates respectively, and the index *T* denotes various tissues. 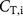 denotes the concentration of chemical *i* in tissue compartment *T*. 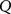 denotes vascular flow rate into tissues, 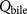 denotes biliary flow rate, 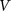 denotes tissue volumes, and 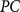 denotes the tissue-blood partition coefficients. The kinetic rate constants, *K*, are explained in Table 1.
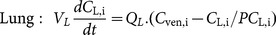
(1)

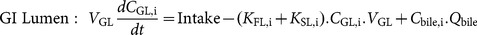
(2)

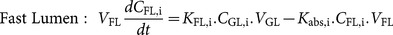
(3)

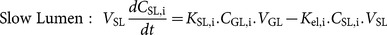
(4)

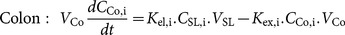
(5)

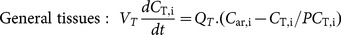
(6)

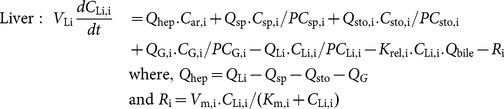
(7)

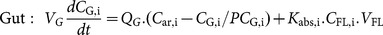
(8)

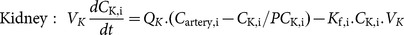
(9)

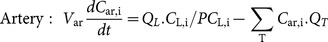
(10)

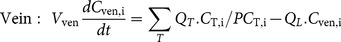
(11)

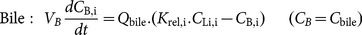
(12)

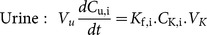
(13)

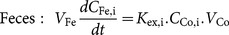
(14)


**Table 1:** Physiological parameters in PBTK model for different species (per min).

Relevant Processes (rats)	Parameter	ZEA	ZOL	GLU
Gastric absorption	*K*_abs_	0.1545	0.1545	0.0833
Fecal excretion	*K*_ex_	0.0115	0.0115	0.053
Urinary filtration	*K*_f_	0.0016	0.0002	0.0013
Biliary recirculation	*K*_rel_	0.0076	0.0203	0.2596

Relevant Processes (humans)	Parameter	ZEA	ZOL	GLU
Absorption	*K*_abs_	15.45	15.45	8.33
Slow luminal absorption	*K*_SL_	0.05	0.05	0.05
Fast luminal absorption	*K*_FL_	20	20	20
Elimination	*K*_el_	3.45	3.45	15.9
Fecal excretion	*K*_ex_	0.0004	0.0004	0.0004
Bile recirculation	*K*_rel_	0.76	2.03	25.96
Urinary filtration	*K*_f_	0.0016	0.00025	0.99

### Parameter estimation

The tissue:blood partition coefficients (PC) for ZEA are taken from Shin *et al.* [34]. The coefficients for ZOL were partly taken from Haritova *et al.* [35] and partly calculated by the same method as in Haritova *et al.*, using the algorithm developed by Poulin & Theil [36] in addition to the tissue composition data (Table S1 in S1 Information) from Poulin & Theil [36]. The PCs for GLU were calculated using the same algorithm employing the octanol:water PC of tetrahydrocannabinol glucuronide, which has similar molecular weight and characteristics to those of ZEA and ZOL-glucuronides. Details of the calculation method have been included in S1 Information. The computed PCs are summarised in Table 2. In the “rat version” of the present model, metabolism of compounds is assumed to take place only in the liver, since extra-hepatic metabolism is negligible in rats [37]. For estimation of physical parameters for absorption, and elimination, the 12 compounds considered in the model have been grouped into 3 classes based on their structural and chemical similarities. The PBTK model consists of four parameters related to absorption and elimination. They are 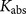 (rate constant for gastric absorption into the gut from the gastrointestinal (GI) lumen), 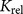 (rate constant for transfer from liver into bile), 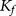 (rate constant for filtration from blood to urine) and 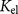 (rate constant for elimination from GI lumen into feces). The rate constant for intestinal absorption of ZEA was estimated by Ramos *et al.* [38] to be 9.27 per hour. The absorption of the glucuronide is expected to be considerably less as it is expected to be preferentially excreted in feces. An *apriori* value for the absorption rate constant for GLU was assumed to be 5 per hour, which was later optimized. Migdalof *et al.* [16] estimated the total radioactivity in feces after ZEA oral dosage in multiple species. The *in vivo* data were utilized along with analysis of fecal radioactivity by Fitzpatrick *et al.* [17], who estimated proportions of ZEA, ZOL and GLU in feces. Kinetic parameters for fecal excretion for the 3 classes of compounds were thus estimated utilizing intestinal amounts from Ramos *et al.* [38] and the fecal data described before. The relevant data has been summarized in Tables S2, S3, S4, & S5 in S1 Information. Subsequent to metabolism, some of the metabolites, as well as unmetabolized components, are released by the liver into biliary secretion. For rats, biliary excretion forms the major route of ZOL and ZEA excretion [16]. The temporal kinetics of biliary radioactivity from Migdalof *et al.* [16] and the component concentrations in rat liver from the same source were utilized to estimate the rate constant for biliary recirculation for all three classes of compounds. The relevant data has been summarized in Tables S6, S7, S8, S9, & S10 in S1 Information. The rate constant for the rate of ultrafiltration between blood and urine in kidney was estimated from the urine radioactivity analysis by Migdalof *et al.* [16] and the blood radioactivity at same time points. The relevant data has been summarized in Tables S11, S12, S13, S14, S15, S16, S17, & S18 in S1 Information. Table 1 summarizes the estimated parameter values. Michaelis-Menten kinetics were assumed for the metabolic processes. The reaction rate constants represent the kinetics for the net reactions as potential reverse reactions are difficult to identify and quantify *in vivo*. The values of the kinetic constants are summarized in Table 3. The exact kinetics of the reactions catalysed by various enzymes involved in the metabolism have not been completely investigated yet, though Malekinejad *et al.* [21] studied the activities of various forms of the HSD enzyme in the catalysis of zearalenone reduction to alcohol. Pfeiffer *et al.* [26] studied the activities of various UGTs for the glucuronidation of compounds of the zeranol family. The activity data has been summarized in Table S19 in S1 Information. Malekinejad *et al.* [21] studied the amounts of various metabolites in hepatic microsomes after allowing them to react with different initial concentrations of zearalenone. The Michaelis-Menten parameters were fitted to the data for *in vivo* conversion of ZEA and ZOL collected by Malekinejad *et al.* [21]. The estimated values of the kinetic parameters are summarized in Table 3.

**Table 2:** Tissue-to-blood partition coefficients for 3 classes of compounds.

Tissue	ZEA	ZOL	GLU
Lung	2.31	6.35	0.73
Liver	4.56	5.57	0.66
Spleen	0.81	2.94	0.58
Kidneys	5.55	5.09	0.67
Heart	1.16	4.31	0.64
Testes	0.49	0.40	0.51
Brain	1.06	13.27	1.04
Muscle	0.43	3.15	0.57
Adipose	3.31	8.46	0.14
Stomach	1.30	8.06	0.78

**Table 3:** Biochemical reaction rate constants for PBTK models.

	*V*_max_/*K_m_* for rat	*V*_max_/*K_m_* for human
Reaction	(ml/min per g liver)	(ml/min per g liver)
ZEA → *α*-ZOL	2.36×10^−2^ [21]	2.36×10^−2^ [21]
ZEA → *β*-ZOL	7.057×10^−3^ [21]	7.057×10^−3^ [21]
*α*-ZOL → *α*-ZAL	2.5×10^−5^	2.5×10^−5^
*β*-ZOL → *β*-ZAL	2.5×10^−5^	2.5×10^−5^
*α*-ZAL → ZAN	6×10^−2^	6×10^−2^
*β*-ZAL → ZAN	3×10^−2^	3×10^−2^
ZAN → *α*-ZAL	1.5×10^−3^	1.5×10^−3^
ZAN → *β*-ZAL	1.5×10^−3^	1.5×10^−3^
ZEA → ZAN	5×10^−6^	5×10^−6^
ZEA → ZEAGLU	4.776×10^−3^ [26]	4.776×10^−3^ [26]
*α*-ZOL → *α*-ZOLGLU	4.806×10^−3^ [26]	4.806×10^−3^ [26]
*β*-ZOL → *β*-ZOLGLU	4.005×10^−3^ [26]	4.005×10^−3^ [26]
*α*-ZAL → *α*-ZALGLU	3.224×10^−3^ [26]	3.224×10^−3^ [26]
*β*-ZAL → *β*-ZALGLU	3.224×10^−3^ [26]	3.224×10^−3^ [26]
ZAN → ZANGLU	5.607×10^−3^ [26]	5.607×10^−3^ [26]

### Extension of the PBTK model to human subjects

ZEA and its family of compounds show considerable differences in metabolism and biological activity among various mammalian species. The metabolism of ZEA has been discussed in detail in the “Exposure and Biotransformation” section. Migdalof *et al.* [16] studied the biotransformation of zeranol in the rat, rabbit, dog, monkey and human. This work found taleranol (*β*-zeranol) and zearalanone as the major metabolites apart from the conjugated glucuronides. The extent of conjugation has also been found to have major inter-species variation. Migdalof *et al.* [16] found glucuronidation to be 99% in humans compared to only 1% in dogs. The mode of excretion of zeranol also varies widely among species. Humans excrete zeranol mostly by urine [27], [39] whereas for dog and rat, fecal elimination is the major route of excretion. It was also discussed in the “Excretion” section, that unlike rats where conversion of ZOLs to ZALs was not found to occur, the conversion takes place to some extent in other mammals such as pigs and humans. An additional unique feature of human metabolism of ZAL is that both ZAL and ZEA are metabolized to some extent in the intestine [33]. Any attempt at developing a PBTK model for human subjects must incorporate these inter-species differences and a simple extrapolation of a model developed for rats might not be sufficient. The PBTK model developed here for human subjects uses a similar structure and the same number of physiological compartments as described previously for the PBTK model for rats, but modifies the gastro-intestinal (GI) system for humans to account for GI metabolism (data summarized in Tables S20 & S21 in S1 Information) and the hepatic system to incorporate inter-species differences in metabolism. The PBTK model for human subjects was developed so that the physiological parameters of the individual to be simulated could be supplied as arguments to the model, thus allowing the prediction of biodistribution for an individual or a population easily. The values of partition coefficients are the same as that of the PBTK model for rats. All conjugated glucuronides have been assumed to have the partition coefficients measured by Skopp *et al.* [40]. The other physical constants relating to transport, absorption and distribution of the chemicals in the body have been estimated in the same way as described for the PBTK model for rats in the “Parameter estimation” section, only in this case human *in vivo* data from Migdalof *et al.* [16] was used for the purpose. Details of the calculation have been included in S1 Information. The calculated values of these parameters were used as initial estimates and were optimized according to the comparison of the results with the *in vivo* biomarker results in humans from Migdalof *et al.* [16]. The final values of the physical parameters have been summarized in Table 1. The biochemical parameters (summarized in Table 3) were estimated utilizing *in vitro* measurements with microsomes harvested from various human organs and cultured with ZEA, *α*-ZAL and their metabolites [21], [26]. The parameters were scaled up to particular organ weights of individuals utilizing established *in vitro* to *in vivo* scale up equations for liver [41] and small intestine [42]. The details of the calculation procedure have been included in S1 Information. It is well known that the pig liver is similar in many ways to the human liver and pig liver has in fact been used for transplants in humans with liver failure. So, wherever human biochemical data are missing, the biochemical parameters for the pig liver have been used after scaling up to the human liver.

### Application of Exposure and Toxicokinetic Modeling to The Jersey Girl Study

The Jersey Girl Study (JGS) involved a cohort of peri-pubertal, predominantly white, New Jersey girls 9-11 years old, to evaluate presence and levels of urinary mycotoxins and its impact on pubertal growth and development [43]. Urine collected from the girls (morning voids) were analysed for ZEA and ZAL and their metabolites. Urine was analysed for all six compounds ZEA, *α*-ZOL, *β*-ZOL, *α*-ZAL, *β*-ZAL and ZAN; however no measurements were made for the glucuronidated forms of the chemicals. About 75% of the samples showed detects of at least one metabolite. The JGS also involved the collection of three dietary recalls for each subject, in which detailed information on all food consumed in the corresponding three previous days was recorded. Food consumption was classified into twenty-seven relevant food groups (summarized and explained in Table 4). This section describes how the PBTK model developed and evaluated with rat and human *in vivo* measurements has been implemented for the JGS subjects to “connect” dietary dose of ZEA and *α*-ZAL to the levels of ZEA, ZAL and their metabolites in urine. A detailed dose estimation has been conducted based on information of ZEA contamination in different food items from the scientific literature. Subsequently, a probabilistic dietary exposure assessment was conducted, considering various uncertainties and variabilities in food contamination and consumption by the JGS subjects.

**Table 4:** Major food groups considered in the Jersey Girl Study for analyzing dietary recalls of subjects (WGRN, SWGRN, & RFGRN are considered as grains; SVEG, NSVEG, & OTVEG are considered as vegetables; PORK, LAMB, POULT, & SAUSG are considered as other meats; FISH & SHFISH are considered as seafood).

**BEEF**	Beef	**ORGAN**	Organ
**DAIRY**	Diary Products	**OTMILK**	Other milk products
**EGGS**	Eggs	**OTVEG**	Other vegetables
**FISH**	Fish	**PCORN**	Popcorn
**FRT**	Fruits	**PORK**	Pork
**LAMB**	Lamb	**POULT**	Poultry
**LEG**	Legumes	**RFGRN**	Refined grains
**MEATALT**	Alternative meat products	**SAUSG**	Sausage
**MILK**	Milk	**SHFISH**	Shell fish
**MLSUPP**	Meal supplements	**SWGRN**	Some whole grains
**NDAIRY**	Non-dairy products	**SVEG**	Starchy vegetables
**NDMLSUP**	Non-dairy meal supplements	**WGRN**	Whole grains
**NSVEG**	Non-starchy vegetables	**VEAL**	Veal
**NUTS**	Nuts		

#### Human Exposure Characterization

**Figure 5:**
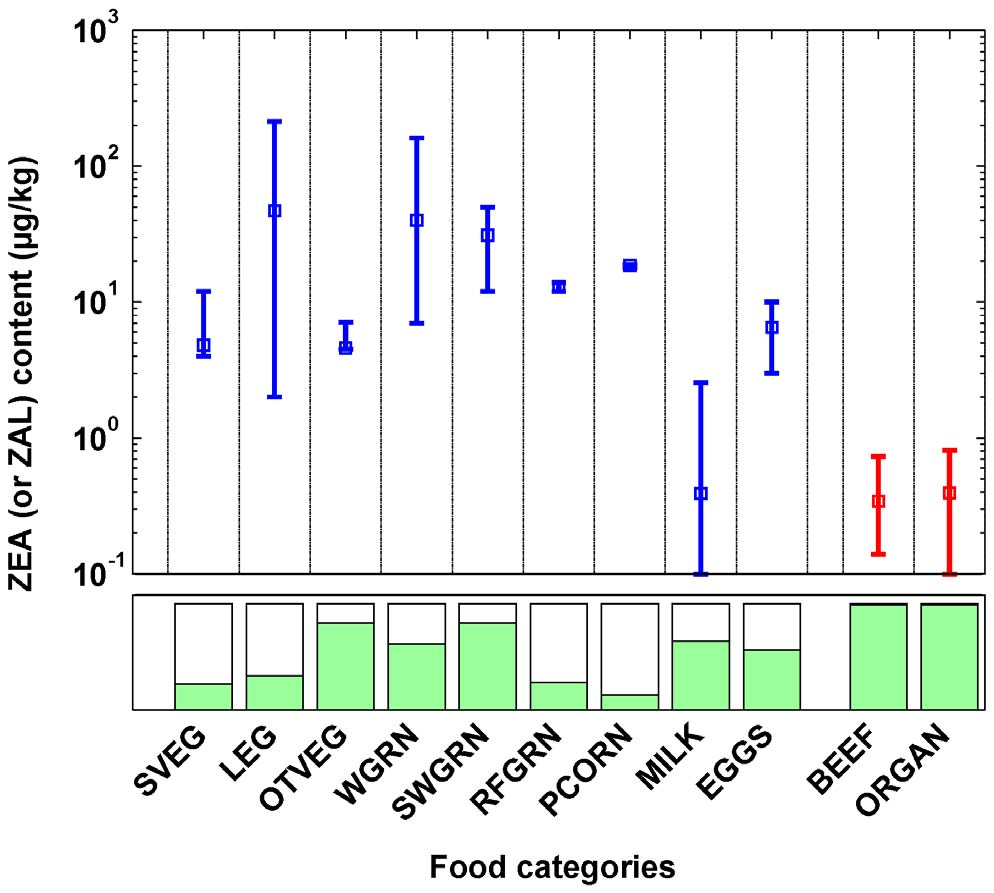
Dietary concentrations. Summary of available dietary concentrations (means and ranges) from published literature of ZEA (in blue) and *α*-ZAL (in red) in major food groups identified for the Jersey Girl Study (Food groups explained in Table 4); green bars at the bottom represent the percentages of positive detects among the food samples tested (Only the food groups with any positive detects identified from the literature are shown here).

Exposure assessment of contaminants must consider multiple uncertainties and variabilities associated with contaminant concentrations in relevant media as well as with human behavior patterns. The MENTOR (Modeling ENvironment for TOtal Risk) framework [44] considers a comprehensive source-to-dose analysis of chemicals of concern which requires detailed, case-specific data. For situations where such detailed data are not available, as in the present study, a screening level system, PRoTEGE (Prioritization/Ranking of Toxic Exposures with GIS Extension) [45] that was developed employing a LCA (Life Cycle Analysis) approach along with basic human LSA (Life Stage Analysis) to identify potential exposures to chemicals of current and emerging concern, for which significant information gaps may exist. In the present study, the PRoTEGE system has been implemented for ZEA and *α*-ZAL using available data on concentrations of ZEA reported in the literature. The analysis can provide an approximate estimate of exposure for a first-tier analysis of the problem at hand. The WHO report of 2000 [10] regarding food additives and contaminants and the European Food Safety Authority (EFSA) report of 2011 [46] included compiled ZEA concentrations in food items from around the world. However, the contamination levels estimated in food vary widely and often have a wide range of values even when taken from the same study. There is large uncertainty in the process, particularly in the variety of geographical origins of different food items. Uncertainty is increased by the fact that the concentration of ZEA in a particular food item is a function of the level of fungal contamination, which in turn is affected by multiple factors such as geographical location, climate (tropical or temperate), availability of storage and transport facilities, duration of storage, use of fungicides, etc. Dietary estimation of ZEA and ZAL has been carried out using scientific literature by prioritizing studies from the continental US and Europe, to ensure similar geographic, climatic, and economic conditions to ones relevant to the JGS. However, since it is very difficult to obtain ZEA concentrations in all the different food items comprising a group, each food group has been represented by a single food item. The contamination levels of ZEA and *α*-ZAL in various food groups obtained from the literature are summarized in Table S22 in S1 Information. The range of concentrations and percent positive detects among the samples tested are utilized to develop a lognormal distribution of mycotoxin concentration. Fig. 5 shows the mean and range of the levels of ZEA and *α*-ZAL in the various food groups as obtained from the literature. Other than the mean and range, the percentage of positive detects (shown as colored bars at the bottom in Fig. 5) among the samples tested has also been taken into consideration while estimating dietary exposure. The percent detects value has been used in a probabilistic model to capture the latent uncertainty in the process of selecting random food samples from the market. ZEA amounts in animal tissues, milk, and eggs have been reported widely in the literature [47]–[49] but most studies have focussed on analyzing ZEA concentrations in animals after various doses of ZEA. Coffey *et al.* [50] recently presented a probabilistic assessment of ZEA levels in milk based on carry over of ZEA from regular feed. the values estimated by Coffey *et al.* have been used in the exposure model. Kleinova *et al.* [29] reported ZEA concentrations in muscle and liver of cows fed regular feed to be negligible. Consequently, ZEA concentration in meat from cows raised on regular feed has been assumed to be zero. Concentrations of *α*-ZAL in beef have been obtained from studies [49] which used doses of *α*-ZAL comparable to common *Ralgro* doses used in commercial meat production. However, these studies all found some concentrations of *α*-ZAL in beef. Exposure to *α*-ZAL from beef consumption would depend on the time gap between date of application of the *Ralgro* implant in the particular animal and the date of consumption. Also, organic beef in the market is assumed to be free of synthetic hormones or additives. Accordingly, the market share of organic beef in the US market [51] was used to estimate the probability of not being exposed to *α*-ZAL due to beef consumption.

#### Dose estimation

An important factor in the implementation of the PBTK model to the JGS is the development of realistic estimates of the daily dietary dose received by the subjects from their food. In this study, available information on the dietary habits of the subjects have been used. These include food consumption data of the JGS subjects, based on three dietary recalls of the subjects which was classified into twenty relevant food groups. However, the day of the recalls range from the day prior to urine collection to three or more days prior to urine collection, with some recalls being for days post-urine collection. Without exact information on food consumed on each day prior to urine collection, it is very difficult to accurately reconstruct the dietary dose. To take into account the multiple uncertainties in levels of food contamination and dietary information, a probabilistic dietary exposure module has been developed here using the range of ZEA/ZAL contamination levels obtained from the literature. In this analysis, individual JGS subjects are denoted by the *i* and the twenty food groups by *j*. The serving amounts consumed by the subjects are randomly selected from the three dietary recalls available for each subject, in order to capture intra-individual variability in food intake. Serving amount, 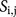, for subject 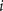 and food group 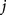 is used along with a Monte Carlo sampled concentration value from a probability distribution of food contamination levels. A log-normal probability distribution, 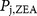 is fitted to each food group 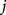 using range of ZEA concentrations obtained from the literature. 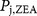 is then combined with a distribution of percent detects, 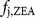, for that food group. We define 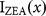 as an indicator function applied on 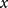, a uniform random variable on [0,1] as:
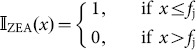
(15)


The probability distribution for daily dose for food group *j*, and subject *i*, 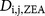 can then be calculated as:
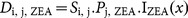
(16)


Similar equations apply to *α*-ZAL also using the range of concentrations for *α*-ZAL from the literature. A random value of daily dose is sampled via Monte Carlo method from the distribution 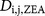 for every simulation, summed up over all food groups to obtain the daily exposed dose, 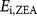 of ZEA as:
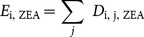
(17)


**Figure 6:**
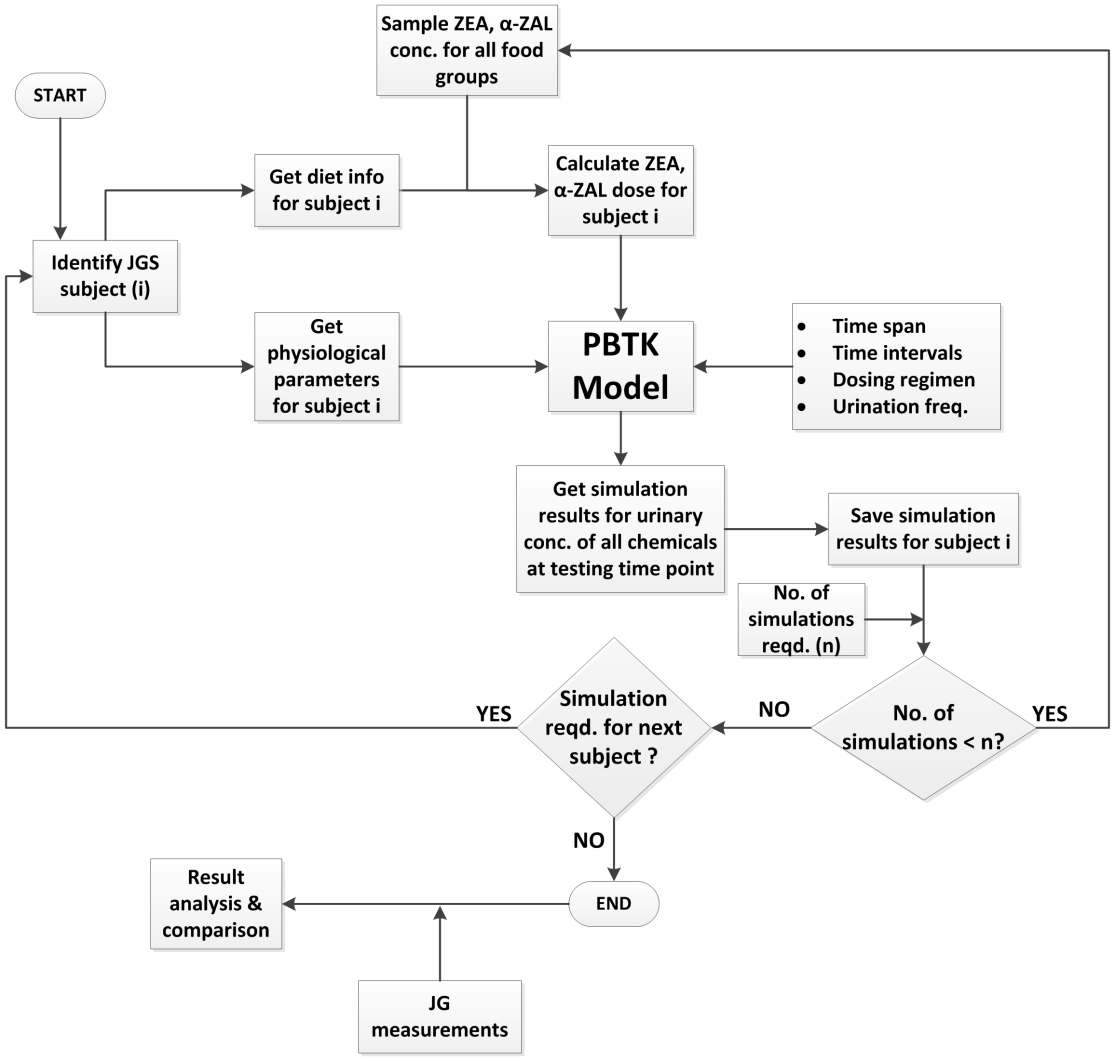
Flowchart for model implementation and simulation. Flowchart showing steps comprising dose estimation and PBTK model simulation for subjects of the Jersey Girl Study.

The daily dose estimated above is divided into three equal intake doses corresponding to three meals every day at 7 AM, 12 noon, and 7 PM. The intakes estimated from this module are used as inputs to the PBTK model simulations for each individual, employing the process outlined in Fig. 6.

## Results

The PBTK model developed here was implemented for a set of case studies and the results are compared with *in vivo* results available from multiple literature sources and for multiple species. The various implementations are outlined here as representative case studies. The ordinary differential equation (ODE) based models developed for this study were coded in MATLAB Release 2013a (Mathworks, Natick, Massachusetts, USA); the same software platform was used to perform all simulations, data analysis, and plotting of results.

### Case study 1 - Implementation for rats

**Figure 7:**
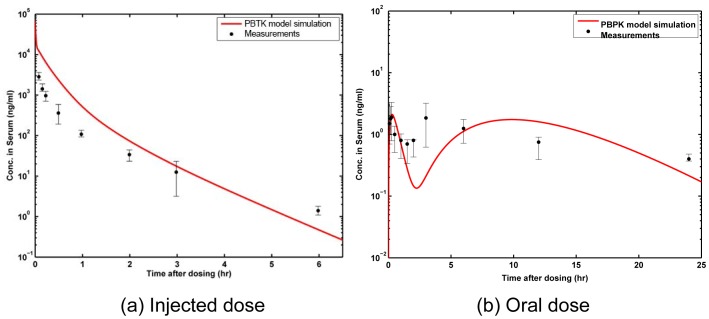
Model predictions for rats. Blood serum (venous blood) concentration of ZEA for 8 mg/kg BW of (a) injected dose, and (b) oral dose over a period of 24 hours; PBTK model predictions (red line) compared with *in vivo* measurements in rats from Shin *et al.* [34].

The PBTK model developed for rats was implemented for 3 different dosage routes in 8–10 week old Sprague-Dawley rats and the model predictions over time were compared with *in vivo* measurements in rats from Shin *et al.* [31], [34]. Intravenous (IV) injection presents the least complicated situation with respect to biodistribution of a chemical. Partitioning of the chemical between various tissues and blood, and excretion from the body through various routes can be studied without the complexities of gastro-intestinal absorption. Data from *in vivo* IV injection studies by Shin *et al.* [34] were used to parametrize and evaluate the PBTK model. Using the data, all biochemical parameters except for those relevant to the GI tract were estimated. Comparisons of results for this dosage route are shown for venous blood serum in Fig. 7(a). Additional results for various other tissues and comparisons for multiple IV doses are included in S1 Information (Figure S2). The kinetics of ZEA in blood rapidly increases instantaneously after the IV dose and then decreases exponentially as also observed in the *in vivo* measurements. The study also focussed on the effects of oral ingestion of ZEA and ZAL in rats. The ZEA oral ingestion study was evaluated with data from Shin *et al.* [34], where Sprague-Dawley rats were given a single dose (8 mg/kg body weight) by *po* gavage. Fig. 7(b) shows the venous concentration of ZEA in rats after the single oral dose over a 24 hour period compared with *in vivo* data from Shin *et al.*. The model successfully captures the two consecutive peaks in venous blood concentration subsequent to oral dosage. The secondary peak in venous concentration after oral dosage was reported previously in multiple species [31], [52], [53] and may be due to biliary recirculation or temporary storage in tissues and subsequent vascular recirculation. The *in vivo* data for rats helps to evaluate the model and build confidence for its use as a tool for planning and validating future *in vivo* experiments. The model also provides initial estimates for various biochemical parameters for the PBTK model for human subjects for which *in vivo* data are not easily available. The model was further evaluated with *in vivo* data from an intravenous infusion study [31], to compare steady-state and long-term dynamics of the PBTK model developed for rats. Results are presented in S1 Information (Figure S3) for comparisons with two different rates of infusion.

### Case study 2 - Implementation for human subjects

**Figure 8:**
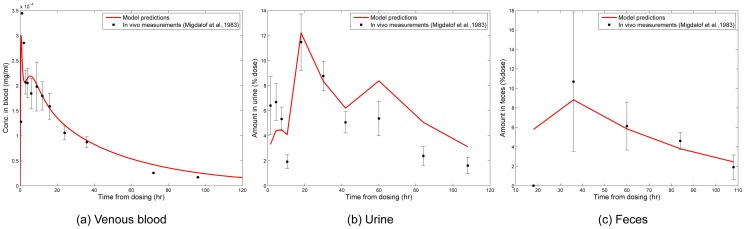
Model predictions for adult human subjects. Total chemicals in (a) venous blood, (b) urine, and (c) feces after ingestion of 1.23 mg/kg body weight of *α*-ZAL by adult healthy human subjects: results from PBTK model (red line) compared with *in vivo* measurements from Migdalof *et al.* [16].

The results from the PBTK model for human subjects were compared with *in vivo* measurements from Migdalof *et al.* [16]. Accordingly, the model was run with the physiological parameters of a 61 kg human which is the mean body weight of the human subjects studied by Migdalof *et al.* Physiological parameters were extracted from the NHANES database [54] corresponding to body weights of subjects. Migdalof *et al.* presents total radioactivity amounts in venous blood, feces and urine. The amounts of individual chemicals in the PBTK model results were added to reflect the total biomarker levels in urine and feces. Fig. 8 compares model predictions with measurements from Migdalof *et al.* [16], showing good agreement of model estimates with observations. However, data for comparing model results for other metabolites are not available. Migdalof *et al.* have analysed biomarker levels for individual chemicals, but only briefly, stating the fraction of zeranol and metabolites which are free or conjugated.

### Case study 3 - Application to the Jersey Girl Study

**Figure 9:**
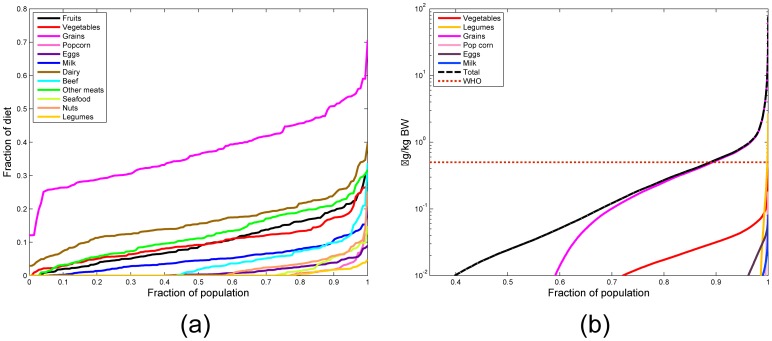
Dietary contributions to exposure. Contributions of the various major food groups towards (a) entire diet of the JGS subjects and (b) estimated ZEA dose; major food groups were constituted by combining similar smaller food groups as described in Table 4.

**Figure 10:**
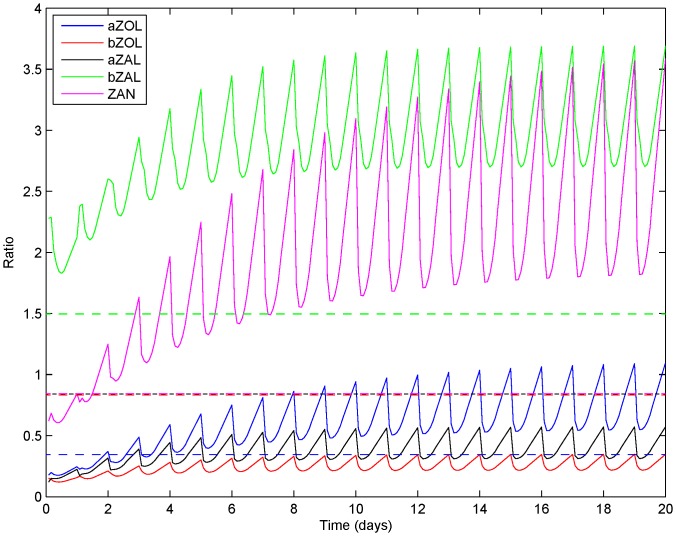
Metabolites in urine. Ratios of various metabolite amounts predicted in urine with respect to ZEA amounts over 20 days of daily intake (Mean values of JGS measurements shown as horizontal dashed lines).

**Figure 11:**
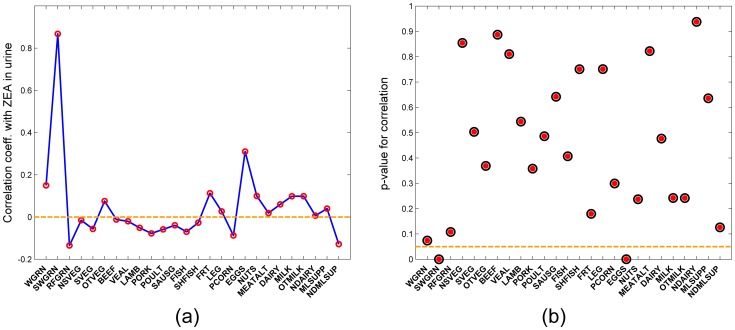
Biomarker-to-food correlation. (a) Correlation coefficients of median ZEA concentrations in urine predicted for the JGS cohort with number of servings of food consumed from various food groups and (b) Corresponding p-values for the correlation coefficients (Dotted line in figure (b) corresponds to a significance level of 0.05) (Food groups explained in Table 4).

**Figure 12:**
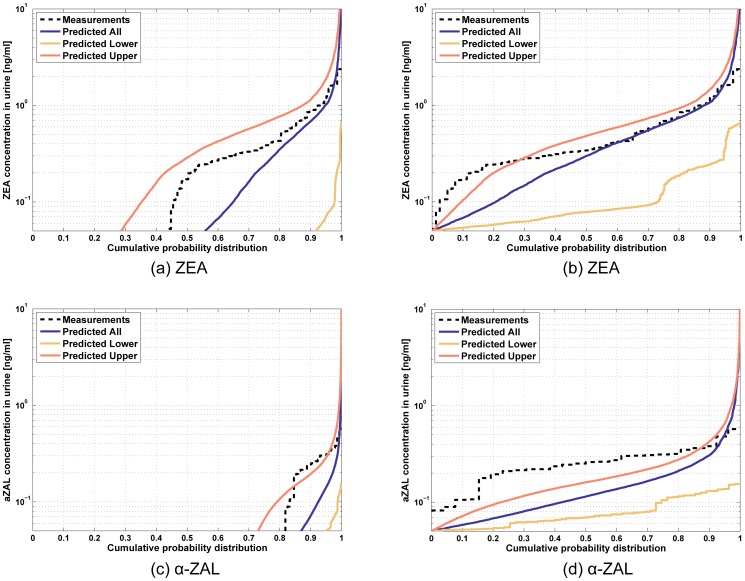
Urinary biomarker predictions. Predicted probability distributions corresponding to higher, lower, and median dietary exposure estimates for the entire cohort of 143 girls for urinary biomarker levels compared with measured values; (a), (c) distributions shown with measured percentages of non-detects in the population, and (b), (d) distributions representing only the samples with detects.).

The PBTK model was implemented for the JGS subjects using physiological parameters obtained from the NHANES database [54] commensurate with the age and the recorded body weight of each subject. The steps in this implementation are summarized schematically in Fig. 6. Daily intakes estimated as discussed in the “Dose estimation” section, were used as inputs to the model as bolus doses. Fig. 9 shows a cumulative probability distribution function of ZEA doses received from various major food groups with comparison to the ADI proposed by WHO [10], showing that the study population might receive a dose greater than the WHO proposed ADI only about 10% of the time. Model simulations were conducted 100 times for each subject of the JGS, to capture variability and uncertainty in dose estimation. It was found that the levels of chemicals in the body became steady after a period of approximately 20 days. Accordingly, the simulations included 20 additional days prior to the urine collection date. A realistic urination frequency was considered [55] including accepted sleeping and waking periods. The predicted urinary concentrations of the chemicals were corrected for urine dilution by specific gravity using the formula by Pearson *et al.* [56] and using the measured specific gravity in morning void urinary samples of female children [56]. Fig. 10 shows the model predictions for the levels of ZEA metabolites in urine for a span of 20 days, for a virtual subject (with average body weight of the JGS cohort) normalized to levels of the parent compound ZEA. Means of observations from the JGS are represented by horizontal dotted lines for the five metabolites. Correlation coefficients were estimated for ZEA and *α*-ZAL amounts predicted in urine with number of servings of food consumed by the subjects. Overall, both ZEA and *α*-ZAL were found to be positively correlated with amount of food consumed (Table 5) with sufficiently low p-values. The positive detects in urine for ZEA and *α*-ZAL were also found to be positively correlated with the measured observations for corresponding subjects (Table 5), though the value for *α*-ZAL is associated with a high p-value signifying that the association is probably less significant. Fig. 11(a) shows correlation coefficients of predicted ZEA amounts in urine with food consumption from all the major food groups considered in the JGS. Whole grains and eggs show the highest correlations with increased ZEA in urine, and the high correlations are also accompanied by low (<0.05) p-values as shown in Fig. 11(b). The entire collection of correlation coefficients for different food groups are summarized in detail in Table S23 in S1 Information. Fig. 12 shows probability distribution predictions for ZEA and *α*-ZAL in urine for the entire cohort of JGS subjects compared with corresponding measurements. ZEA and *α*-ZAL intake distributions were calculated for each subject separately for three ranges - high, low, and medium. These three intake ranges were used to predict urinary biomarker distributions corresponding to high, low, and medium exposure levels of JGS subjects.

**Table 5:** Correlations of predicted ZEA and *α*-ZAL concentrations in urine.

Chemicals	With total number of servings	With measured concentrations
ZEA	0.1616 (p = 0.0538)	0.4045 (p = 0.0036)
*α*-ZAL	0.2421 (p = 0.0036)	0.0257 (p = 0.9276)

For developing the biomarker probability distribution, results of multiple simulations for each subject from the PBTK model were combined, taking into account the proportions of non-detects similar to the JGS measurements. Fig. 12 shows the three predicted levels based on the Monte Carlo sampled doses from the three exposure levels. The left panel of figures (Figs. 12(a) and 12(c)) show the predicted distributions considering the same proportions of non-detects as in the JGS cohort, while the right panel (Figs. 12(b) and 12(d)) show corresponding predictions only for the positive detects. The prediction for the median exposure level agrees well with the measurements and the measurements are always within the bounds of the high and low prediction levels. For *α*-ZAL (Figs. 12(c) and 12(d)), the measured amounts are closer to the predicted lower bounds for most of the values. This might be due to *α*-ZAL exposures of the JGS subjects being lower than those estimated from dietary analysis. Fig. 10 compares the urinary biomarker levels corresponding to the five metabolites of ZEA with measured values in the JGS subjects.

## Discussion

In summary, the work presents the development and application of a comprehensive PBTK model for zearalenone (ZEA) and its metabolites, including *α*-zeranol which is used as an additive in beef in the US and Canada, and is the first model known to consider all major metabolites and their glucuronides. The model was implemented for rats and for human subjects and evaluated successfully with comparison of blood, urinary, and fecal biomarkers from published *in vivo* studies. The model was also applied to a case study involving a potentially vulnerable population of 9–11 year old girls in New Jersey, USA, using a comprehensive probabilistic dietary exposure estimation and dose analysis. Subject-specific and population-wide comparison of model predictions and urinary measurements were presented. Population-wide probabilistic estimates corresponding to high, low and median dietary intake estimates are presented together, that capture inter and intra-individual variability and also agree well with measured values for the predicted high and low expected exposure levels. Overall, the measured values and model predictions are of the same order of magnitude despite huge uncertainties in diet, meal times, and food sources used in the calculations. The predicted ZEA concentrations in urine were found to be positively correlated (0.1616, p = 0.054) with amounts of food consumed, especially with the consumption of grains (Fig. 11) as part of the diet. Grains also form a major portion of the total diet in the subjects of the JGS (as shown in Fig. 9), which also drives the increased exposure to ZEA. Blood elimination half-lives were also estimated for the cohort of young girls in the JGS. For calculating blood elimination, a single dose of ZEA or *α*-ZAL was used in the modeled individuals and the half-life of elimination of parent compound and its metabolites was calculated from the model predictions. The half-life for ZEA was estimated to be 11.89 (±1.51) hours for the JGS subjects, and that for *α*-ZAL was 11.94 (±1.97) hours (summarized in Table 6). The value is lesser than the value reported by Migdalof *et al.* [16] in adult human subjects, which is probably due to the lower body weights and consequently smaller volume of distribution in the young girls as compared to adult humans. The half-life for ZEA in the JGS subjects is lower than that estimated in rats (Shin *et al.* [34]) due to the fact that ZEA kinetics after oral ingestion are predicted to be faster in humans than in rats. Humans with appreciable entero-hepatic recirculation coupled with intestinal metabolism would be expected to metabolize ZEA faster than rats, which have no intestinal metabolism.

**Table 6:** Blood elimination half-lives of zearalenone and zeranol.

Chemical	Subject (avg. body weight)	Half-life (in hrs.)	Reference
ZEA	Male Sprague-Dawley rats (0.26 kg)	16.8±8.4	Shin *et al.* [34]
*α*-ZAL	Adult human subjects (61 kg)	22	Migdalof *et al.* [16]
ZEA	Young human female subjects (35.75 kg)	11.89±1.51	Current study
*α*-ZAL	Young human female subjects (35.75 kg)	11.94±1.97	Current study

### Model uncertainty and sensitivity

**Figure 13:**
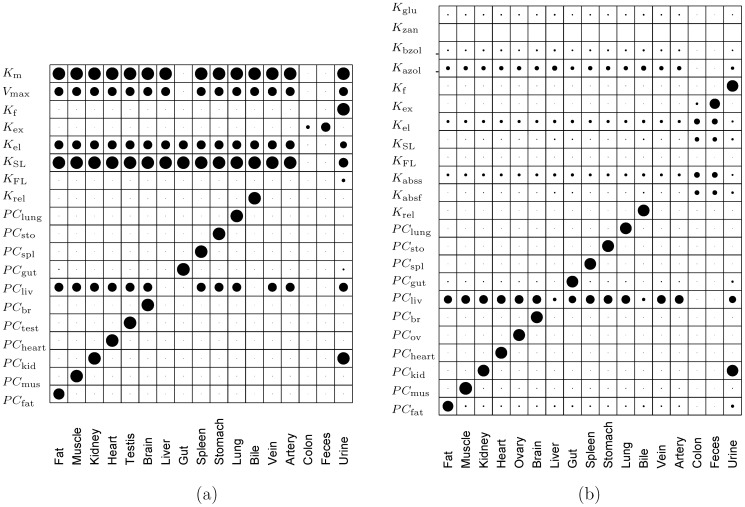
Sensitivity analysis of model. Calculated sensitivity indices for PBPK model implementation in (a) rats and (b) human subjects based on oral dosage. Both models were tested with an oral bolus dose of 8 mg/kg BW of ZEA and ZEA amounts in tissues after 12 hours were used to calculate the sensitivity indices. The size of dots shown in the figures represents the relative sensitivity of each parameter compared to all other aspects of the model.).

Model predictions of human biomarker levels for zearalenone and zeranol are affected by (a) variability in model parameter values estimated for human populations and (b) uncertainties in dietary exposure estimation. Parameter estimation for human populations cannot be conducted directly as done for other mammals and therefore parameters are generally obtained through *in vitro-in vivo* extrapolation of measurements from *in vitro* cultures (discussed in detail in the “Parameter estimation” & the “Extension of the PBTK model to human subjects” sections). The model utilizes *in vitro* and *in vivo* data available in the scientific literature regarding chemical kinetics of zearalenone and its metabolites. However, there is still wide variability within these data related to species tested, *in vitro* cultures used, and chemicals evaluated. This causes variability in model estimates, which could be potentially improved by performing improved measurements of these compounds and their metabolites using human tissue samples *in vitro*. Consequently, a key step towards developing better models is to identify important parameters that most substantially affect the final results, and subsequently design future studies for obtaining improved estimates of those key parameters. Results of sensitivity analyses conducted on the parameters of the PBPK models that were developed for the rats and the human subjects in this work, are presented in Fig. 13. Fig. 13 shows the sensitivity indices as black dots with the relative size of the dots signifying a proportionally greater or lesser relative sensitivity. Sensitivity indices have been calculated according to the following equation:
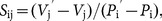
(18)where 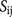 is the sensitivity index for parameter 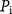 based on output variable *V*_j_. Here, 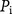 and 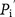 are respectively, the original value and changed value of the *i*th parameter. 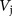 and 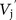 are the corresponding changes in the *j*th output variable due to the change in parameter value. The calculated sensitivity indices are then normalized based on their maximum value. Fig. 13(a) shows that the PBPK model results for rats are highly sensitive in general to kinetic parameters 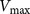 and 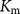 which control metabolism of ZEA in the liver. ZEA concentrations in the tissues are sensitive to the corresponding partition coefficient (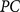) for that tissue, except 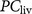 which affects whole body distribution of ZEA. The model is also especially sensitive to the parameters for gut elimination (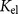) and that for slow absorption in the gut lumen (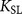). This is expected since, for oral dosage, gut absorption, elimination, and hepatic metabolism are key processes controlling ZEA concentration in tissues. Key parameters for urinary biomarker levels include the hepatic metabolic parameters (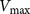, 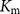), filtration parameter (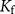), and the partition coefficients of liver and kidney (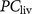, 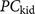). The PBPK model implementation for human subjects (Fig. 13(b)) shows a similar high sensitivity for the tissue partition coefficients corresponding to the respective tissue concentration. Additionally, higher sensitivity is observed for the kidney filtration parameter and kidney partition coefficient, especially for urinary biomarker levels. Overall, it can be stated that the liver and kidney appear to be key controlling compartments affecting the whole-body distribution, metabolism and biomarker levels in both rats and humans. Better estimation of parameters pertaining to key compartmental processes is important in addressing observed large uncertainties associated with model predictions. A better characterization of these parameters in the future, will also help capture inter-individual variations in whole body distribution and metabolism of the compounds of concern.

## Summary

The work presented here introduces a method for estimating population-wide exposures, intakes, and biodistribution kinetics for ZEA, ZAL, and their metabolites, providing important predictions for potentially vulnerable populations, that can be used to determine estrogenic effects in individuals over a long span of time. The work is the first of its kind to link a comprehensive PBTK model for zeranol and its metabolites in humans, with a comprehensive exposure assessment and biomarker comparison in vulnerable human subjects. Quantification of levels of critical mycoestrogens and their metabolites in various tissues of the human body over time can help estimate potential estrogenic effects in vulnerable populations over extended periods of time. Of specific interest is their residence and storage in adipose tissue and their effect on the human endocrine system, eventually leading to endocrine disruption and potential carcinogenic events. The model implementation for a vulnerable population of pre-pubertal girls in addition to a comprehensive dietary dose estimation using dietary recalls, is a considerable step towards source-to-dose-to-effect prediction of xenobiotic estrogenic compounds.

## Supporting Information

S1 Information **Details of parameter estimation and some additional results.**
(DOCX)
