# Supplementary material for: Physiologically-Based Toxicokinetic Modeling of Zearalenone and Its Metabolites: Application to the Jersey Girl Study
Source: PLoS One. 2014 Dec 4;9(12):e113632. doi: 10.1371/journal.pone.0113632 (PMC4256163; doi:10.1371/journal.pone.0113632)
Supplement: S1 Information — Details of parameter estimation and some additional results. (DOCX) [file pone.0113632.s001.docx]

**Supplementary information for :**

**Physiologically-Based Toxicokinetic Modeling of Zearalenone and its Metabolites: Application to the Jersey Girl Study**

Dwaipayan Mukherjee, Steven G. Royce, Jocelyn A. Alexander, Brian Buckley,
Sastry S. Isukapalli, Elisa V. Bandera, Helmut Zarbl, and Panos G. Georgopoulos

**Zearalenone (ZEA) metabolism pathways**

**
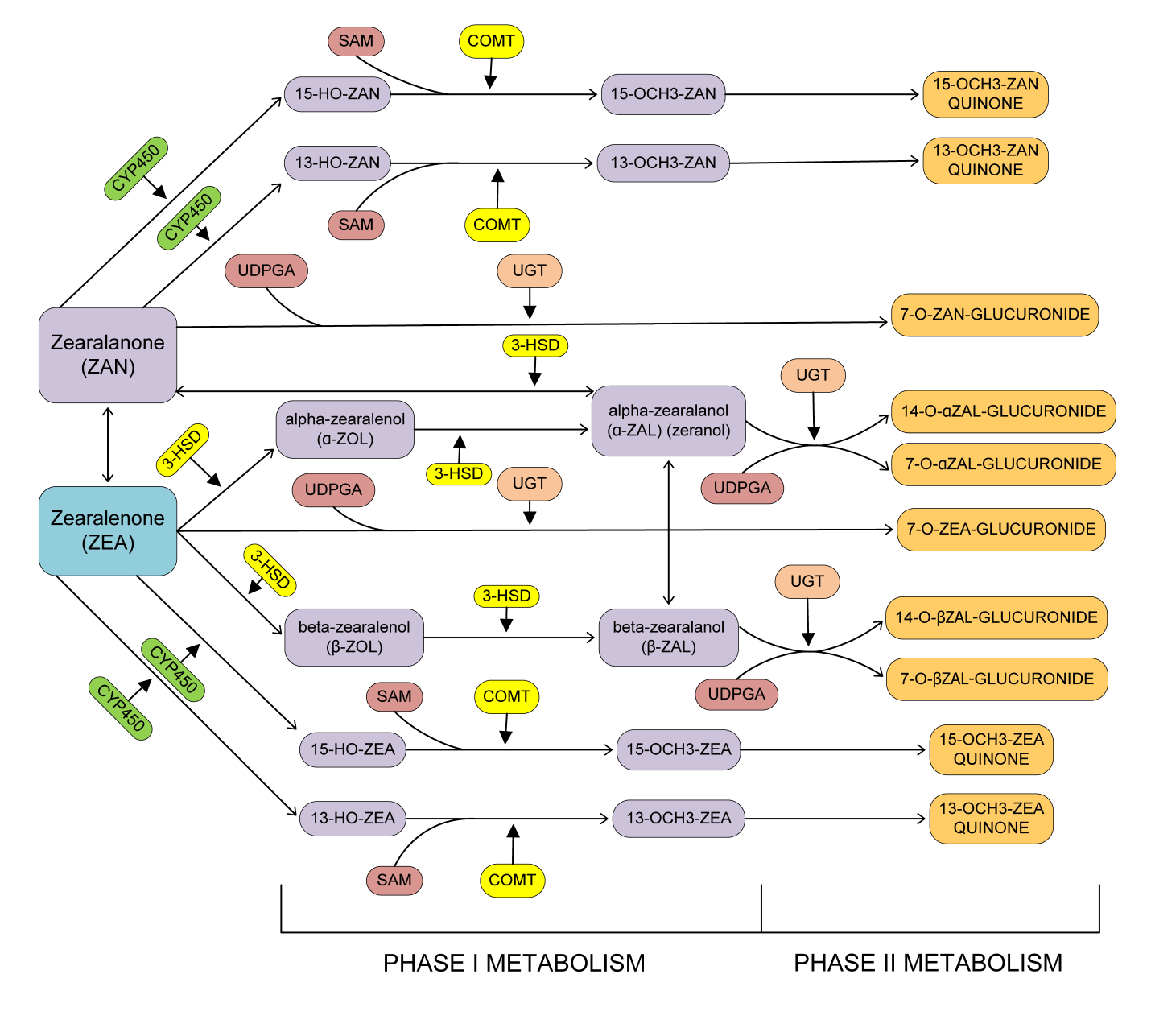
**

Figure S1: Phase I and Phase II metabolism pathways for ZEA

**Estimation of partition coefficients**

Tissue : Plasma partition coefficients, *P_t:p_* are estimated according to Poulin & Theil^(1)^ as:

$$P_{t:p}=\frac{\left[P_{\mathrm{o}:w}\left(V_{nlt}+0.3V_{pht}\right)\right]+\left[V_{wt}+0.7V_{pht}\right]}{\left[P_{\mathrm{o}:w}\left(V_{nlp}+0.3V_{php}\right)\right]+\left[V_{wp}+0.7V_{php}\right]}\times\frac{fu_{p}}{fu_{t}}$$

*P_o:w_* = octanol:water partition coefficient (for non-adipose tissues)

= olive oil:water partition coefficient (for adipose tissue)

*f_up_* = unbound fraction of chemical in plasma

*f_ut_* = unbound fraction of chemical in tissue ( = 1 for adipose tissue)

*V_nl_* = fractional volume of neutral lipids

*V_ph_* = fractional volume of phospholipids

*V_w_* = fractional volume of water

Table S1. Tissue composition data for estimation of partition coefficients

| **Tissues** | **Water (*V_w_*)** | **Neutral Lipids (*V_nl_*)** | **Phospholipids (*V_ph_*)** |
| --- | --- | --- | --- |
| Adipose | 0.12 | 0.853 | 0.002 |
| Brain | 0.788 | 0.0392 | 0.0533 |
| Small Intestine | 0.749 | 0.0292 | 0.0138 |
| Heart | 0.779 | 0.014 | 0.0118 |
| Kidney | 0.771 | 0.0123 | 0.0284 |
| Liver | 0.705 | 0.0138 | 0.0303 |
| Lung | 0.79 | 0.0219 | 0.014 |
| Muscle | 0.756 | 0.01 | 0.009 |
| Spleen | 0.771 | 0.0077 | 0.0136 |
| Stomach | 0.748 | 0.0292 | 0.0138 |
| Testes | 0.861 | 0.00033 | 0.0024 |

**Tissue composition data from Poulin & Theil^(1)^; Data for testes from Davis *et al*.^(2)^; Data for stomach from Reinoso *et al.^(3)^***

For zeranol, log *PC_oc:w_* = 3.13 (at pH = 7) (Schering-Plough, 1998^(4)^)

So, *PC_oc:w_* = 1348.96

*fu_p_* for zeranol was estimated to be 0.156 using data estimated for zeranol by Haritova *et al.*^(5)^

*fu_t_* was calculated using the relation: $fu_{t}=\frac{1}{1+0.5\left(\frac{1-fu_{p}}{fu_{p}}\right)}=\frac{2fu_{p}}{1+fu_{p}}$

Partition coefficients (PC) for all glucuronides were estimated using the equation described before and the tissue composition data summarized in Table S1.The octanol:water PC for tetrahydro-cannbinol glucuronide ( = 17.4 [Skopp *et al* ^(6)^]) was used. The olive oil:water PC was estimated using the relation between *PC_oc:w_* and *PC_o:w_* given by Poulin & Theil^(1)^ for propranolol:

$$\frac{PC_{oc:w}}{PC_{o:w}}=\frac{3.2}{0.164}=19.51$$

Using the same ratio, *PC_o:w_* for GLU = 17.4/19.51 = 0.892

**Estimation of physical parameters**

## Fecal excretion parameters in PBTK model for rats and humans

(Main data from Migdalof, *et al.* ^(7)^)

$$\begin{matrix} \frac{dm_{G}}{dt}=-k_{a}.m_{G}-k_{el}.m_{G}, k_{a}=\text{absorption} \text{constant} \text{(Fitzpatrick,1988)} \\ \frac{dm_{c}}{dt}=k_{el}.m_{G}-k_{ex}.m_{c}, k_{el}=\text{elimination} \text{constant (to be estimated)} \\ \frac{df}{dt}=k_{ex}.m_{c}, k_{ex}=\text{excretion} \text{constant (to be estimated)} \end{matrix}$$

Here we consider the 2 chemicals ZOL and GLU independently. We assume no interconversion between them in the gut and colon and so each chemical is absorbed independently and is also excreted. The absorption constants, *k_a_* have been estimated from the percentage of total dose excreted in urine or feces by Fitzpatrick *et al*.^(8)^.

Table S2. Fecal amounts of ZEA and its metabolites for rats

| Time (hr.) | Feces (% dose) (cumulative) | GLU* | ZOL* |
| --- | --- | --- | --- |
| 12 | 0.39 | 0.312 | 0.0176 |
| 24 | 28.49 | 22.792 | 1.2821 |
| 48 | 64.69 | 51.752 | 2.9111 |
| 72 | 71.55 | 57.24 | 3.2198 |
| 96 | 73.88 | 59.104 | 3.3246 |

*Calculated from percentages (80% GLU, 15.5% ZEA, 4.5% ZOL) reported by Fitzpatrick *et al.* ^(8)^

The above set of differential equations has been solved by changing parameters and comparing the results with the above tabulated measurement results. The parameter values have been optimized using the *MATLAB* subroutine *fminsearchbnd.*  The estimated parameters are summarized in the table below.

For ZEA, however, we have only the 96 hour value as reported by Fitzpatrick *et al*.^(8)^. Comparing with that single value, and using the parameters of ZOL as first guesses, we get:

Table S3. Parameter values for fecal excretion for rats

| Parameter | ZEA | ZOL | GLU |
| --- | --- | --- | --- |
| Elimination from colon | 0.0034 | 0.0003 | 0.0113 |
| Excretion in feces | 0.0013 | 0.0002 | 0.0003 |

For humans, the same method is applied using human data from Fitzpatrick *et al*.^(8)^

Table S4. Fecal amounts of ZEA and its metabolites for humans

| Time (hr.) | Feces (% dose) (cumulative) | ZOL* | GLU* |
| --- | --- | --- | --- |
| 24 | 0.02 | 0.0009 | 0.016 |
| 48 | 10.09 | 0.45405 | 8.072 |
| 72 | 16.22 | 0.7299 | 12.976 |
| 96 | 20.83 | 0.93735 | 16.664 |
| 120 | 22.75 | 1.02375 | 18.2 |

*Calculated from percentages (80% GLU, 15.5% ZEA, 4.5% ZOL)
reported by Fitzpatrick *et al*.^(8)^

The above set of differential equations has been solved by changing parameters and comparing the results with the above tabulated measurement results. The parameter values have been optimized using the MATLAB function *fminsearchbnd.*  The estimated parameters are summarized in the table below.

For ZEA however, we have assumed the same value as estimated for the rat, because of absence of human data.

Table S5. Parameter values for fecal excretion for humans

| Parameter | ZEA | ZOL | GLU |
| --- | --- | --- | --- |
| Elimination from colon | 0.0034 | 6.375×10^-5^ | 0.0142 |
| Excretion in feces | 0.0013 | 2.418×10^-4^ | 4.088×10^-5^ |

## Biliary recirculation parameter in PBTK model for rats and humans

(Main data from Migdalof, *et al*.^(7)^)

$$\begin{matrix} C_{liv}=C_{blood}.PC_{liv:blood} \\ \frac{dm_{b}}{dt}=k_{b}.C_{liv}.V_{liv}, k_{b}=\text{biliary} \text{recirculation} \text{constant} \end{matrix}$$

Table S6. Blood concentrations in rats

| Time (hr.) | Blood (conc./dose) | GLU* | ZOL* |
| --- | --- | --- | --- |
| 0.5 | 0.07 | 0.0559 | 0.0141 |
| 1 | 0.034 | 0.0271 | 0.0069 |
| 2 | 0.025 | 0.02 | 0.005 |
| 4 | 0.024 | 0.0192 | 0.0048 |
| 8 | 0.018 | 0.0144 | 0.0036 |
| 12 | 0.013 | 0.0104 | 0.0026 |
| 24 | 0.011 | 0.0088 | 0.0022 |

*Migdalof *et al*^(9)^, reports about 80% of the concentration in blood and urine to be conjugated

Results from curve-fitting (blood concentration):

$$\text{ZOL:} C_{blood}=(0.0082)t^{-0.434} \text{(\% dose,} \text{hr.)}$$

$$\text{GLU:} C_{blood}=(0.0328)t^{-0.434} \text{(\% dose,} \text{hr.)}$$

Table S7. Liver – Blood partition coefficients for rats

| ZEA | ZOL | GLU |
| --- | --- | --- |
| 4.56 | 5.5702 | 0.6562 |

Converting blood concentration to liver concentration using partition coefficients:

$$\text{ZOL:} C_{liv}=(0.0457)t^{-0.434} \text{(\%dose,} \text{hr.)}$$

$$\text{GLU:} C_{liv}= (0.0215)t^{-0.434} \text{(\%dose,} \text{hr.)}$$

Table S8. Biliary recirculation amounts in rats

| Time (hr.) | Bile (% dose) | GLU* | ZOL* |
| --- | --- | --- | --- |
| 2 | 8.5 | 6.8 | 0.425 |
| 4 | 13.8 | 11.04 | 0.69 |
| 8 | 26.2 | 20.96 | 1.31 |
| 12 | 35.8 | 28.64 | 1.79 |
| 24 | 70.7 | 56.56 | 3.535 |

* Assuming 80% GLU and 5% ZOL according to Migdalof *et al*^(7)^.

Results from curve-fitting:

$$\begin{matrix} \text{ZOL:} \text{(\% dose,} \text{hr.)} \\ m=(0.1441)t+0.0906 \\ dm/dt=0.1441 \end{matrix}$$

$$\begin{matrix} \text{GLU:} \text{(\%dose,} \text{hr.)} \\ m=(2.3061)t+1.4492 \\ dm/dt=2.3061 \end{matrix}$$

$$k_{b}=\frac{1}{C_{liv}.V_{liv}}.\frac{dm_{b}}{dt}\times\frac{1}{100}, k_{b}=\text{biliary recirculation} \text{constant}$$

The value of the filtration rate constant will be a function of time. We find the average over time for each chemical. The values are presented in the table below. The value of the parameter for ZEA is estimated from Shin *et al.*^(10)^, where they have estimated 2 parameters for biliary recirculation and biliary elimination. We have added these 2 parameters as we have considered both these processes together in bile flow in our PBTK model. The average amount of ZEA in the liver as predicted by the Shin model is about 0.5 ng/g. We have used that information to get the fraction of ZEA recirculated into bile per min.

Table S9. Final estimated values of *k_b_* for rats

| ZEA (min^-1^) | ZOL (min^-1^) | GLU (min^-1^) |
| --- | --- | --- |
| *0.00086 | 0.0083 | 0.283 |

*after scaling with body weight

Due to absence of biliary data in humans, we have assumed the biliary parameters calculated in rats for humans. The parameter for ZEA as estimated from Shin *et al.*^(10)^ was scaled by body weight and is different due to a different body weight for humans (70 kg).

Table S10. Final estimated values of *k_b_* for humans

| ZEA | ZOL | GLU |
| --- | --- | --- |
| *0.0585 | 0.0083 | 0.283 |

*after scaling with body weight

## Urinary filtration parameters in PBTK model for rats and humans

## (Main data from Migdalof, *et al*. ^(7)^)

$$\begin{matrix} C_{kid}=C_{blood}.PC_{kid:blood} \\ \frac{dm_{u}}{dt}=k_{f}.C_{kid}.V_{kid}, k_{f}=\text{urinary} \text{filtration} \text{constant} \end{matrix}$$

Results from curve-fitting (blood concentration) as shown in last section:

$$\text{ZOL:} C_{blood}=(0.0082)t^{-0.434} \text{(\% dose,} \text{hr.)}$$

$$\text{GLU:} C_{blood}=(0.0328)t^{-0.434} \text{(\% dose,} \text{hr.)}$$

Table S11. Kidney – Blood partition coefficients in rats

| ZEA | ZOL | GLU |
| --- | --- | --- |
| 5.55 | 5.0894 | 0.6729 |

Converting blood concentration to kidney concentration using partition coefficients:

$$\text{ZOL:} C_{kid}=(0.0417)t^{-0.434} \text{(\% dose,} \text{hr.)}$$

$$\text{GLU:} C_{kid}= (0.022)t^{-0.434} \text{(\% dose,} \text{hr.)}$$

Table S12. Urinary amounts in rats

| Time (hr.) | Urine (% dose) (cum.) | GLU* | ZOL* |
| --- | --- | --- | --- |
| 12 | 2.83 | 2.023 | 0.0708 |
| 36 | 5.21 | 3.725 | 0.1303 |
| 48 | 6.79 | 4.855 | 0.1698 |
| 72 | 7.39 | 5.284 | 0.1848 |
| 96 | 7.53 | 5.384 | 0.1883 |

*based on 71.5% GLU and 2.5% ZOL in rat urine according to Mirocha *et al*. ^(11)^

Results from curve-fitting:

$$\begin{matrix} \text{ZOL:} \text{(\% dose,} \text{hr.)} \\ m=-(2\times10^{-5})t^{2}+(0.004)t+0.0256 \\ dm/dt=-(4\times10^{-5})t+0.004 \end{matrix}$$

$$\begin{matrix} \text{GLU:} \text{(\% dose,} \text{hr.)} \\ m=-(7\times10^{-4})t^{2}+(0.1132)t+0.7308 \\ dm/dt=-(1.4\times10^{-3})t+0.1132 \end{matrix}$$

$$k_{f}=\frac{1}{C_{kid}.V_{kid}}.\frac{dm_{u}}{dt}\times\frac{1}{100}, k_{f}=\text{urinary} \text{filtration} \text{constant}$$

The value of the filtration rate constant will be a function of time. We find the average over time for each chemical. The values are presented in Table S15.

For ZEA, we use data from Mirocha *et al*. ^(11)^. They do not report blood concentrations, so we used the blood concentration data from Shin *et al*.^(10)^ extrapolating for the dose used by Mirocha *et al*. ^(11)^, assuming linear dose-response. They have data only for the entire urine collected after 96 hours. So we try optimizing the urinary parameter to match the total urinary excretion after 96 hrs.

Table S13. Kidney-blood partitioning of chemicals in rats

| Time (hr.) | Blood conc.(ng/ml)  (Shin *et al*.)  (dose = 2mg) | Estimated blood conc. (ng/ml) for Mirocha *et al*. (dose = 5mg) | Estimated kidney conc.  (k:b partition coeff. = 5.55) |
| --- | --- | --- | --- |
| 0.08 | 1.5 | 3.75 | 20.8125 |
| 0.17 | 1.8 | 4.5 | 24.975 |
| 0.25 | 1.9 | 4.75 | 26.3625 |
| 0.5 | 1 | 2.5 | 13.875 |
| 1 | 0.8 | 2 | 11.1 |
| 1.5 | 0.7 | 1.75 | 9.7125 |
| 2 | 0.8 | 2 | 11.1 |
| 3 | 1.85 | 4.625 | 25.6688 |
| 6 | 1.25 | 3.125 | 17.3438 |
| 12 | 0.75 | 1.875 | 10.4063 |
| 24 | 0.4 | 1 | 5.55 |
| 96* | 0.15* | 0.385* | 2.134* |

*extrapolated to 96 hrs.

$$\frac{dm_{u}}{dt}=k_{f}.C_{kid}.V_{kid} \Rightarrow m_{u}=\int_{0}^{96} k_{f}.C_{kid}.V_{kid}.dt$$

We can find the total amount of urine excreted in 96 hrs. by integrating as shown by the above equation. The integral may be estimated by finding the area under the curve defined by the above data points. Mirocha *et al*.^(11)^ estimates the final urinary concentration to be 13.7 µg/ml after 96 hrs. of collection. It is estimated that in 96 hrs the total amount of urine produced by a rat is about 96 ml (Rat Phenome Database ^(12)^). So total ZEA obtained in urine after 96 hrs. = 1.315 mg. We optimize the parameter so that the estimated total excretion of ZEA matches the experimentally determined value by Mirocha *et al*. ^(11)^.

Table S14. Final values of the urinary filtration parameter in rats

| ZEA | ZOL | GLU |
| --- | --- | --- |
| 0.00063 | 0.0006 | 0.033 |

For humans, we use data from Migdalof *et al*.^(7)^

Table S15. Blood concentrations in humans

| Time (hr.) | Blood (conc./dose) | ZOL* | GLU* |
| --- | --- | --- | --- |
| 0.5 | 0.104 | 0.00104 | 0.10296 |
| 1 | 0.28 | 0.0028 | 0.2772 |
| 2 | 0.232 | 0.00232 | 0.22968 |
| 3 | 0.168 | 0.00168 | 0.16632 |
| 4 | 0.167 | 0.00167 | 0.16533 |
| 6 | 0.15 | 0.0015 | 0.1485 |
| 9 | 0.161 | 0.00161 | 0.15939 |
| 12 | 0.146 | 0.00146 | 0.14454 |
| 16 | 0.129 | 0.00129 | 0.12771 |
| 24 | 0.086 | 0.00086 | 0.08514 |
| 36 | 0.071 | 0.00071 | 0.07029 |
| 72 | 0.021 | 0.00021 | 0.02079 |
| 96 | 0.014 | 0.00014 | 0.01386 |

*Migdalof *et al* ^(7)^, reports about 99% of the concentration in blood and urine to be conjugated

Results from curve-fitting (blood concentration):

$$\text{ZOL:} C_{blood}=(0.0021)e^{-0.03t} \text{(\% dose,} \text{hr.)}$$

$$\text{GLU:} C_{blood}=(0.2054)e^{-0.03t} \text{(\% dose,} \text{hr.)}$$

Table S16. Kidney – Blood partition coefficients in humans

| ZEA | ZOL | GLU |
| --- | --- | --- |
| 5.55 | 5.0894 | 0.6729 |

Converting blood concentration to kidney concentration using partition coefficients:

$$\text{ZOL:} C_{kid}=(0.0107)e^{-0.03t} \text{(\% dose,} \text{hr.)}$$

$$\text{GLU:} C_{kid}= (0.1382)e^{-0.03t} \text{(\% dose,} \text{hr.)}$$

Table S17. Urinary amounts in amounts

| Time (hr.) | Urine (% dose) (cumulative) | ZOL* | GLU* |
| --- | --- | --- | --- |
| 3 | 6.41 | 0.0641 | 6.3459 |
| 6 | 13.1 | 0.131 | 12.969 |
| 9 | 18.44 | 0.1844 | 18.2556 |
| 12 | 20.37 | 0.2037 | 20.1663 |
| 24 | 31.85 | 0.3185 | 31.5315 |
| 36 | 40.62 | 0.4062 | 40.2138 |
| 48 | 45.69 | 0.4569 | 45.2331 |
| 72 | 51.07 | 0.5107 | 50.5593 |
| 96 | 53.46 | 0.5346 | 52.9254 |
| 120 | 55.08 | 0.5508 | 54.5292 |

*based on 99% GLU and 1% ZOL in human urine according to Migdalof *et al*. ^(7)^

Results from curve-fitting:

$$\begin{matrix} \text{ZOL:} \text{(\% dose,} \text{hr.)} \\ m=(0.1434)\log t-0.1204 \\ dm/dt=(0.1434).\frac{1}{t} \end{matrix}$$

$$\begin{matrix} \text{GLU:} \text{(\% dose,} \text{hr.)} \\ m=(14.198)\log t-11.923 \\ dm/dt=(14.198).\frac{1}{t} \end{matrix}$$

$$k_{f}=\frac{1}{C_{kid}.V_{kid}}.\frac{dm_{u}}{dt}, k_{f}=\text{urinary} \text{filtration} \text{constant}$$

The value of the filtration rate constant will be a function of time. We find the average over time for each chemical. The values are presented in Table S19.

For ZEA, due to non-availability of human feeding experiments, we have assumed the same urinary filtration constant as that estimated for the rat.

Table S18. Final values of the urinary filtration parameter for humans

| ZEA | ZOL | GLU |
| --- | --- | --- |
| 0.00063 | 0.00007 | 0.0055 |

**Parameter estimation for biochemical parameters**

Table S19. Activities of various enzymes involved in metabolism of ZEA and its related compounds in humans (Pfeiffer *et al*.^(9)^)

| **Parent compound** | **Reaction** | **Enzyme** | **Activity (mol/min/mol protein)** |
| --- | --- | --- | --- |
| **ZEN** | Aromatic hydroxylation | CYP1A2 | 12 |
|  |  | CYP3A4 | 1 |
|  | Glucuronidation | UGT1A1 | 0.231 |
|  |  | UGT1A3 | 0.0220 |
|  |  | UGT1A7 | 0.0132 |
|  |  | UGT1A8 | 0.1595 |
|  |  | UGT1A9 | 0.033 |
|  |  | UGT1A10 | 0.0055 |
|  |  | UGT2B15 | 0.00825 |
| **α-ZOL** | Aromatic hydroxylation | CYP1A2 | 16.5 |
|  |  | CYP3A4 | 2 |
|  | Glucuronidation | UGT1A1 | 0.286 |
|  |  | UGT1A3 | 0.099 |
|  |  | UGT1A8 | 0.0385 |
|  |  | UGT1A9 | 0.011 |
|  |  | UGT2B7 | 0.0165 |
| **β-ZOL** | Glucuronidation | UGT1A1 | 0.165 |
|  |  | UGT1A3 | 0.132 |
|  |  | UGT1A8 | 0.187 |
|  |  | UGT1A9 | 0.0385 |
|  |  | UGT1A10 | 0.0055 |
|  |  | UGT2B4 | 0.0055 |
|  |  | UGT2B7 | 0.0165 |
| **ZAN** | Glucuronidation | UGT1A1 | 0.198 |
|  |  | UGT1A3 | 0.077 |
|  |  | UGT1A8 | 0.0825 |
|  |  | UGT1A9 | 0.0275 |
|  |  | UGT2B4 | 0.033 |
|  |  | UGT2B15 | 0.044 |
| **α-ZAL** | Aromatic hydroxylation | CYP1A2 | 50 |
|  |  | CYP3A4 | 6 |
|  | Glucuronidation | UGT1A1 | 0.1595 |
|  |  | UGT1A3 | 0.0605 |
|  |  | UGT1A4 | 0.0055 |
|  |  | UGT1A8 | 0.0165 |
|  |  | UGT1A9 | 0.0275 |
|  |  | UGT2B4 | 0.011 |
|  |  | UGT2B7 | 0.011 |
|  |  | UGT2B17 | 0.011 |
| **β-ZAL** | Glucuronidation | UGT1A1 | 0.066 |
|  |  | UGT1A3 | 0.0605 |
|  |  | UGT1A8 | 0.0275 |
|  |  | UGT1A9 | 0.022 |
|  |  | UGT2B4 | 0.011 |
|  |  | UGT2B7 | 0.0275 |

Amount of microsomal proteins = 14.1 mg/g liver (Malekinejad *et al*.^(13)^)

These values was used to scale the amounts of proteins for the whole liver and the rate constants for the biochemical reactions were estimated by taking a weighted average of the activities of the enzymes listed in Table S21.

Table S20. Michaelis-Menten parameters for intestinal metabolism in humans (Videmann *et al*.^(14)^)

| Compound | V_max_ (pmol/h) | K_m_ (µM) |
| --- | --- | --- |
| ZEA | 8056 | 579 |
| α-ZOL | 208 | 46.9 |
| β-ZOL | 260.5 | 42.9 |
| ZEA glucuronide | 40.1 | 23.6 |
| α-ZOL glucuronide | 53.5 | 10.8 |

The exact kinetics of the catalysis of various enzymes involved in the metabolism has not been completely investigated yet. Pfeiffer et al.^(9)^ studied the activities of various CYP enzymes for the aromatic hydroxylation of ZEN and α-ZOL^(9)^ as well as the activities of various UGTs for the glucuronidation of various compounds of the zeranol family^(15)^.

Table S21. Equilibrium conversion for glucuronidation (Stevenson *et al*.^(16)^)

| Compound | Equilibrium conversion |
| --- | --- |
| ZEN | 0.85 |
| α-ZEL | 0.61 |
| β-ZEL | 0.61 |
| α-ZAL | 0.12 |
| β-ZAL | 0.62 |
| ZAN | 0.82 |

**Zearalenone/α-Zeranol contamination in food**

Table S22. ZEA and α-ZAL (in red) contamination levels in various food groups summarized from the published literature

| **Food group** | **Description** | **Location of food source** | **ZEA/α-ZAL conc. range (µg/kg)** | **Fraction of samples with +ve detects** | **References** |
| --- | --- | --- | --- | --- | --- |
| **SVEG** | Starchy vegetables | EU | 0 - 50 | 45/137 | EFSA, 2011^(17)^ |
| **LEG** | Legumes | Germany | 2 - 214 | 7/45 | Schollenberger *et al*., 2007^(18)^ |
| **OTVEG** | Other vegetables | EU | 0 - 7.1 | 67/100 | EFSA, 2011^(17)^ |
| **WGRN** | Whole grains | EU | 0 - 2969 | 41/100 | EFSA, 2011^(17)^ |
| **SWGRN** | Some whole grains | Michigan, USA | 12 - 50 | 2/3 | Abouzied *et al*., 1991^(19)^ |
| **RFGRN** | Refined grains | Michigan, USA | 12 - 14 | 2/17 | Abouzied *et al*., 1991^(19)^ |
| **PCON** | Popcorn | Canada | 18 - 19 | 2/36 | Kuiper-Goodman *et al*., 1987^(20)^ |
| **MILK** | Milk | EU | 0 - 2.58 | 164/370 | Coffey *et al*., 2009^(21)^ |
| **BEEF** | Beef | UK | 0.14 - 0.73 | 1 | Dixon *et al*., 1986^(22)^ |
| **ORGAN** | Beef organs | UK | 0.1 - 0.81 | 1 | Dixon *et al*., 1986^(22)^ |
| **EGGS** | Eggs | Belgium | 3 - 10 | 7/20 | Tangni *et al*., 2009^(23)^ |


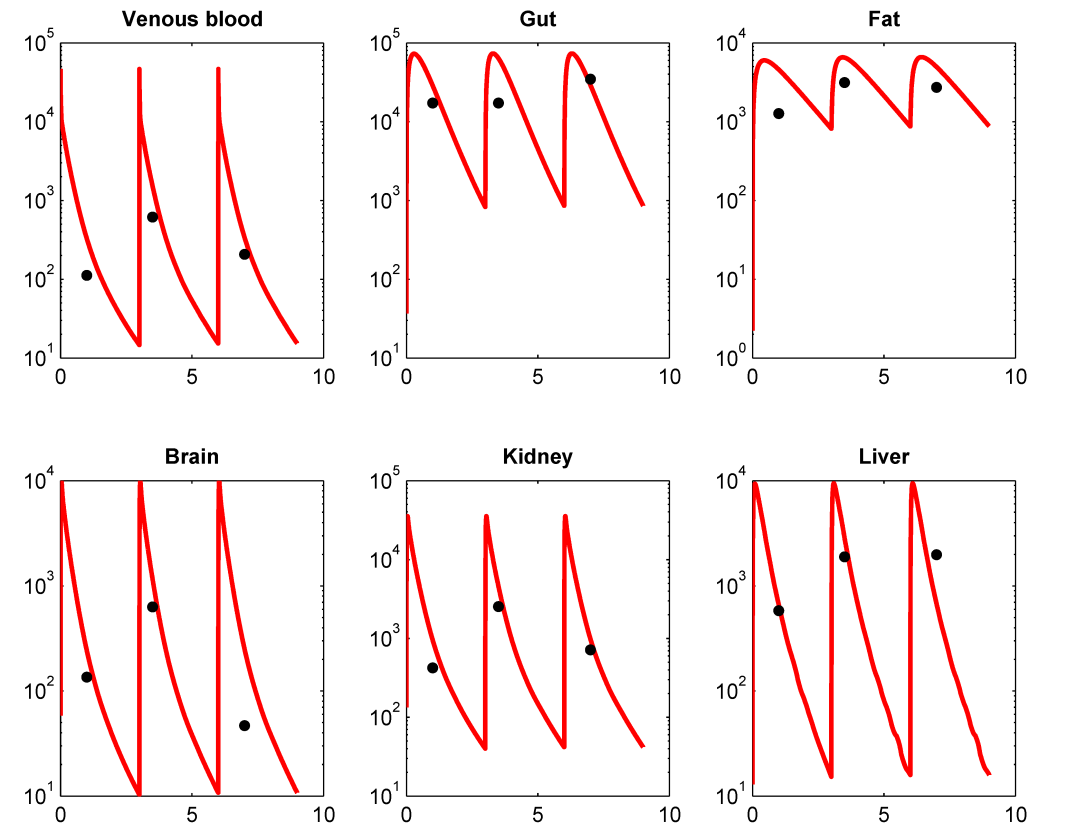
**Additional results for PBTK model evaluation in rats**

Injected multiple dose (8 mg/kg BW) of ZEA once every 3 hours in Sprague-Dawley rats

Figure S2: PBTK model predictions of ZEA concentrations (ng/ml) in various tissues of rats compared with *in vivo* measurements from Shin *et al*. ^(10)^


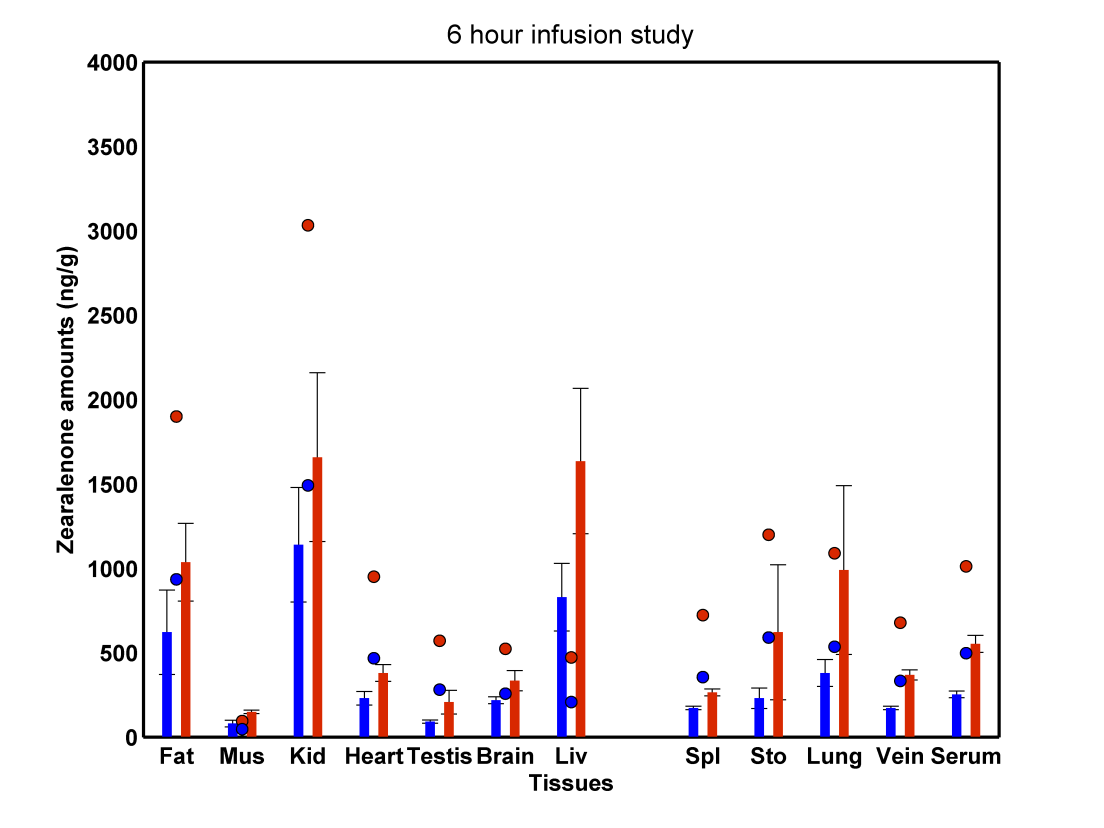


Figure S3: Venous blood amounts of ZEA in rats after 6 hour constant rate infusion into vein at two different rates (bars represent in vivo results from Shin *et al*. ^(10)^ and circles represent PBTK model predictions)

**Additional ZEA and α-ZAL correlation analyses**

Table S23. Correlations of predicted concentrations in urine for the JGS subjects with number of servings in different food groups

| Food groups | Predicted ZEA conc. in urine | | Predicted ZAL conc. in urine | |
| --- | --- | --- | --- | --- |
|  | *R^2^* | *p* | *R^2^* | *p* |
| FRT | 0.1129 | 0.1795 | 0.1143 | 0.1739 |
| NSVEG | -0.0155 | 0.8543 | -0.0317 | 0.7072 |
| SVEG | -0.0564 | 0.5032 | -0.0447 | 0.5964 |
| LEG | 0.0268 | 0.751 | 0.0649 | 0.4409 |
| OTVEG | 0.0757 | 0.3688 | 0.042 | 0.6183 |
| WGRN | 0.1499 | 0.074 | 0.3963 | < 0.0001 |
| SWGRN | 0.8679 | < 0.0001 | 0.8635 | < 0.0001 |
| RFGRN | -0.135 | 0.108 | -0.1754 | 0.0361 |
| PCON | -0.0874 | 0.2994 | -0.1107 | 0.188 |
| BEEF | -0.012 | 0.8869 | -0.0093 | 0.9125 |
| VEAL | -0.0203 | 0.8102 | -0.043 | 0.6105 |
| LAMB | -0.0512 | 0.5437 | -0.0595 | 0.4801 |
| PORK | -0.0775 | 0.3578 | -0.0873 | 0.3 |
| POULT | -0.0587 | 0.486 | -0.0486 | 0.564 |
| FISH | -0.0699 | 0.4069 | -0.0107 | 0.8992 |
| SHFISH | -0.0268 | 0.7506 | -0.032 | 0.7045 |
| SAUSG | -0.0392 | 0.6417 | 0.0402 | 0.6333 |
| ORGAN | - | - | - | - |
| EGGS | 0.3102 | 0.0002 | 0.2931 | 0.0004 |
| NUTS | 0.0995 | 0.2372 | 0.0542 | 0.5203 |
| MEATALT | 0.019 | 0.8221 | 0.0639 | 0.448 |
| DAIRY | 0.06 | 0.4766 | 0.0916 | 0.2764 |
| MILK | 0.0985 | 0.242 | 0.1715 | 0.0406 |
| OTMILK | 0.0985 | 0.2417 | 0.0614 | 0.4666 |
| NDAIRY | 0.0066 | 0.9376 | -0.0116 | 0.8908 |
| MLSUPP | 0.0399 | 0.6359 | 0.0408 | 0.6289 |
| NDMLSUPP | -0.1285 | 0.1261 | -0.1626 | 0.0524 |

**References**

(1) Poulin, P.; Theil, F. P., Prediction of Pharmacokinetics prior to In Vivo studies.II. Generic Physiologically Based Pharmacokinetic Models of Drug Disposition. *Journal of Pharmaceutical Sciences* **2002,** *91*, (5).

(2) Davis, J. T.; Bridges, R. B.; Coniglio, J. G., Changes in lipid composition of the maturing rat testis. *Biochem. J.* **1966,** *98*, (1), 342-346.

(3) Reinoso, R. F.; Telfer, B. A.; Rowland, M., Tissue water content in rats measured by desiccation. *Journal of Pharmacological and Toxicological Methods* **1997,** *38*, (2), 87-92.

(4) Schering-Plough *Environmental Assessment for additional indication for "Improved Feed Efficiency" for Ralgro Magnum ear implants for cattle*; Schering-Plough Animal Health Corp.: Union, New Jersey, 1998.

(5) Haritova, A. M.; Fink-Gremmels, J., A simulation model for the prediction of tissue:plasma partition coefficients for drug residues in natural casings. *The Veterinary Journal* **2010,** *185*, 278-284.

(6) Skopp, G.; Poetsch, L.; Mauden, M.; Richter, B., Partition coefficient, blood to plasma ratio, protein binding and short-term stability of 11-nor-delta9-carboxy tetrahydrocannabinol glucuronide. *Forensic Science International* **2002,** *126*, 17-23.

(7) Migdalof, B. H.; Dugger, H. A.; Heider, J. G.; others, Biotransformation of zeranol: disposition and metabolism in the female rat, rabbit, dog, monkey and man. *Xenobiotica* **1983,** *13*, (4), 209-221.

(8) Fitzpatrick, D. W.; Arbuckle, L. D.; Hassen, A. M., Zearalenone metabolism and excretion in the rat: Effect of different doses. *Journal of Environmental Science and Health* **1988,** *23*, (4), 343-354.

(9) Pfeiffer, E.; Hildebrand, A.; Damm, G.; Rapp, A.; others, Aromatic hydroxylation is a major metabolic pathway of the mycoestrogen zearalenone. *Molecular Nutrition & Food Research* **2009,** *53*, 1123-1133.

(10) Shin, B. S.; Hong, S. H.; others, Physiologically Based Pharmacokinetics of Zearalenone. *Journal of Toxicology and Environmental Health* **2009,** *72*, 1395-1405.

(11) Mirocha, C. J.; Pathre, S. V.; Robison, T. S., Comparative metabolism of zearalenone and transmission into bovine milk. *Food and Cosmetic Toxicology* **1981,** *19*, 25-30.

(12) NBRP The National BioResource Project for the Rat in Japan. <http://www.anim.med.kyoto-u.ac.jp/nbr/Default.aspx> (Accessed 2014 June 20), 2014.

(13) Malekinejad, H.; Maas-Bakker, R. F.; Fink-Gremmels, J., Bioactivation of zearalenone by porcine hepatic biotransformation. *Journal of Veterinary Research* **2005,** *36*, 799-810.

(14) Videmann, B.; Mazallon, M.; others, Metabolism and transfer of the mycotoxin zearalenone in human intestinal Caco-2 cells. *Food and Chemical Toxicology* **2008,** *46*, 3279-3286.

(15) Pfeiffer, E.; Hildebrand, A.; Mikula, H.; Metzler, M., Glucuronidation of zearalenone, zeranol and four metabolites <I>in vitro</I>: Formation of glucuronides by various microsomes and human UDP-glucuronosyltransferase isoforms. *Molecular Nutrition & Food Research* **2010,** *54*, 1-9.

(16) Stevenson, D. E.; Hansen, R. P.; Loader, J. I.; Jensen, D. J.; Cooney, J. M.; Wilkins, A. L.; Miles, C. O., Preparative Enzymatic Synthesis of Glucuronides of Zearalenone and Five of Its Metabolites. *Journal of Agricultural and Food Chemistry* **2008,** *56*, (11), 4032-4038.

(17) EFSA, Scientific Opinion on the risks for public health related to the presence of zearalenone in food. *The EFSA Journal* **2011,** *9*, (6), 2197.

(18) Schollenberger, M.; Mueller, H. M.; Ruefle, M.; Terry-Jara, H.; Suchy, S.; Plank, S.; Drochner, W., Natural Occurrence of Fusarium toxins in soy food marketed in Germany. *International Journal of Food Microbiology* **2007,** *113*, 142 - 146.

(19) Abouzied, M. M.; Azcona, J. I.; Braselton, W. E.; Pestka, J. J., Immunochemical assessment of mycotoxins in 1989 grain foods: evidence for deoxynivalenol (vomitoxin) contamination. *Appl Environ Microbiol* **1991,** *57*, (3), 672-7.

(20) Kuiper-Goodman, T.; Scott, P. M.; Watanabe, H., Risk assessment of the mycotoxin zearalenone. *Regulatory Toxicology & Pharmacology* **1987,** *7*, (3), 253-304.

(21) Coffey, R.; Cummins, E.; Ward, S., Exposure assessment of mycotoxins in dairy milk. *Food Control* **2009,** *20*, (3), 239-249.

(22) Dixon, S. N.; Russell, K. L.; Heitzman, R. J.; Mallinson, C. B., Radioimmunoassay of the anabolic agent zeranol. V: Residues of zeranol in the edible tissues, urine, faeces and bile of steers treated with Ralgro. *Journal of Veterinary Pharmacology and Therapeutics* **1986,** *9*, 353-358.

(23) Tangni, E. K.; Waegeneers, N.; vanOvermeire, I.; Goeyens, L.; Pussemier, L., Mycotoxin analyses in some home produced eggs in Belgium reveal small contribution to the total daily intake. *Science of the Total Environment* **2009,** *407*, (4411 - 4418).
